# Enhancing nanomedicine efficacy in KPC pancreatic tumors through ketotifen-mediated tumor microenvironment remodeling

**DOI:** 10.1016/j.jconrel.2025.114541

**Published:** 2026-02-10

**Authors:** Antonia Charalambous, Fotios Mpekris, Chrysovalantis Voutouri, Constantina Neophytou, José Djamus, Ajay Gupta, Alberto Gabizon, Triantafyllos Stylianopoulos

**Affiliations:** a Cancer Biophysics Laboratory, Department of Mechanical Engineering, University of Cyprus, Nicosia, Cyprus; b Cancer Genetics, Therapeutics & Ultrastructural Pathology Department, The Cyprus Institute of Neurology & Genetics, Nicosia, Cyprus; c AnaBioSi-Data Ltd Company, Nicosia, Cyprus; d The Leah and Jakub Susskind Nano-Oncology Research Laboratory, The Helmsley Cancer Center, Shaare Zedek Medical Center, Jerusalem, Israel; e Faculty of Medicine, The Hebrew University of Jerusalem, Jerusalem, Israel

**Keywords:** Drug Delivery, Tumor normalization, Doxil, PLAD, PDAC

## Abstract

The tumor microenvironment (TME) in pancreatic ductal adenocarcinoma (PDAC) poses major barriers to the efficacy of anticancer therapies, particularly through features such as desmoplasia, elevated stiffness, and impaired perfusion that restrict drug delivery. Strategies that modulate these physical abnormalities hold promise for improving therapeutic outcomes, especially for nanomedicines. In this study, we investigated the potential of the antihistamine ketotifen to remodel the TME and enhance intratumoral delivery and efficacy of liposomal therapeutics, PEGylated liposomal doxorubicin (Doxil), and a co-encapsulated alendronate-doxorubicin (PLAD) formulation. We employed the KPC model of PDAC, a tumor type derived from genetically engineered mouse models that mimic key features of human pancreatic tumors, such as a immunosuppresive microenvironment and dense fibrosis. In vitro, ketotifen demonstrated no direct cytotoxic activity on tumor cells, and did not alter cellular drug uptake of Doxil, PLAD or free doxorubicin as assessed by flow cytometry. *In vivo*, ketotifen pretreatment significantly enhanced intratumoral accumulation of liposomes by reducing stiffness and improving perfusion, as measured by shear wave elastography and contrast-enhanced ultrasound. These microenvironmental changes translated into greater anti-tumor efficacy and prolonged survival in groups that received a combination of ketotifen even at low doses. Cytokine analysis showed reduced IL-10 and an IFN-γ increase in ketotifen combinations, suggesting immune modulation of the TME. Random forest analysis identified tumor stiffness, IL-10, and TNF-α as the strongest predictors of therapeutic outcome. These findings demonstrate that microenvironmental modulation by repurposing ketotifen improves nanomedicine delivery and efficacy in PDAC.

## Highlights

• Ketotifen remodels tumor microenvironment to enhance liposomal accumulation over time.
• Pre-treatment with ketotifen boosts efficacy of the nanomedicines Doxil and PLAD.
• Random forest analysis shows tumor stiffness, IL-10 and TNF-α as key predictors of treatment outcome.

## Graphical abstract

**Figure:**
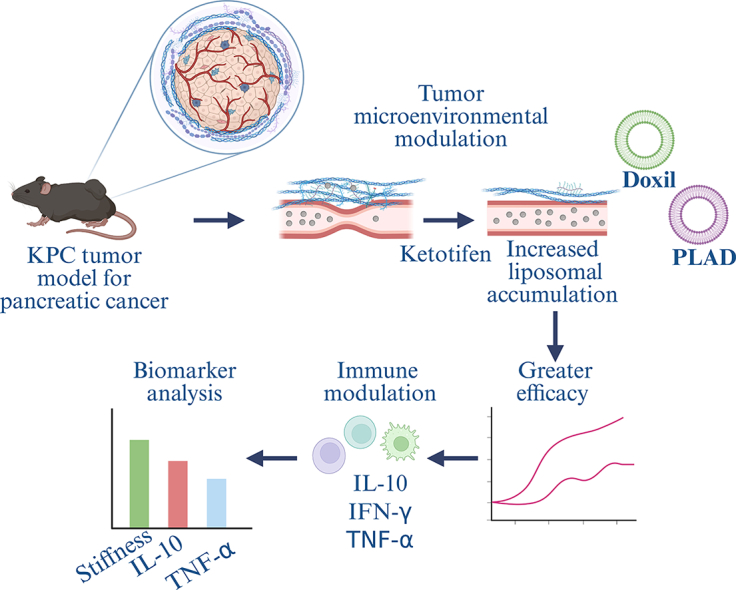

## 1 Introduction

Among the many factors affecting cancer therapy, the tumor microenvironment (TME) stands out as a key regulator of therapeutic success in solid malignancies. The TME is a dynamic and complex network of stromal cells, immune populations, and extracellular matrix (ECM) components that collectively shape tumor progression and response to treatment [1,2]. In desmoplastic tumors, the interplay between cancer cells, stromal cells and the fibrotic ECM – characterized by excessive deposition of collagen and hyaluronan -– leads to tumor stiffening and the accumulation of mechanical forces within the tumor. These two mechanical abnormalities cause compression of tumor vessels, which drastically impairs perfusion, ultimately restricting the delivery and efficacy of therapeutic agents [[3], [4], [5], [6], [7]]. This challenge is particularly evident in pancreatic ductal adenocarcinomas (PDACs) which develop a dense, fibrotic, and therapy-resistant TME with more than 80 % of tumor vessel being collapsed [8,9]. The delivery and homogeneous penetration of nanomedicines (e.g., liposomal and micellar formulations, antibodies, etc.) in such tumors is hindered considerably more than that of low molecular weight drugs due to their relatively large size [10].

A therapeutic strategy to decompress vessels and improve perfusion is the use of mechanotherapeutics to alleviate stiffness and mechanical forces inside the tumors [11]. Mechanotherapeutics are often repurposed drugs (e.g., antihypertensive, antifibrotic and antihistamine drugs) that target the TME by modifying the ECM or reprogramming cancer associated fibroblasts (CAFs). Examples include tranilast, pirfenidone, losartan and ketotifen [[12], [13], [14], [15], [16], [17], [18]]. Among these, losartan was the first drug to be tested in a phase II clinical trial for locally advanced pancreatic tumors. It was found that the addition of losartan to FOLFIRINOX chemotherapy, increased drastically the number of patients eligible for tumor resection from 12 % to 62 % [19]. This success of losartan led to several ongoing multicenter clinical trials in the USA and Italy to further evaluate its efficacy to improve standard-of-care treatment on patients with PDAC (clinicaltrials.gov identifiers: NCT01821729, NCT05077800, NCT05861336, NCT03563248, NCT04106856). Losartan, however, is a potent antihypertensive and patients with hypotension or even normal blood pressure might not be able to tolerate it at the required dose for TME modulation. This was the reason that many patients had to be excluded from the first successful clinical trial. PEGPH20, a nanoparticle formulation of recombinant human hyaluronidase demonstrated promising results in PDAC preclinical studies [20]. It failed, however, in phase 3 trials due to off-target toxicity issues, necessitating significant dose reductions that ultimately proved to be clinically ineffective [21].

The antihistamine ketotifen exerts mechanotherapeutic effects by inhibiting mast cell activation, suppressing CAF proliferation, and reducing ECM synthesis, ultimately leading to remodeling of the TME in preclinical models of sarcomas and breast tumors [15,18,22,23]. We have further shown its efficacy to remodel the TME in patients with sarcoma [15,24]. Given that antihistamines may be safer and better tolerated than antihypertensives or new formulations by patients with cancer, we chose to investigate the ability of ketotifen to remodel the TME in PDAC and improve therapy, an approach which is still largely unexplored.

To this end, we investigated the potential of ketotifen to enhance the delivery and efficacy of nanomedicines in genetically engineered KPC tumors, which is an established and clinically relevant model of human PDAC in mice [25]. The KPC model recapitulates key histopathological and stromal features of human PDAC, including extensive fibrosis, vascular compression and immune cell composition [20,26]. Transcription profiling identified KPC tumors as basal-like, corresponding to the most therapy resistant PDAC subtype [27]. To enable non-invasive monitoring of tumor stiffness and perfusion dynamics during treatment using shear wave elastography and contrast-enhanced ultrasound methods, we used a subcutaneous KPC model, which preserves the ECM and tumor microenvironmental properties of pancreatic tumors [28,29]. Two doxorubicin-based liposomal formulations were employed: PEGylated liposomal doxorubicin (commercially known as Doxil) and a PEGylated liposome co-encapsulating alendronate and doxorubicin (PLAD) [24,[30], [31], [32]]. While PLAD was developed based on the Doxil formulation, it stands out due to its unique combination of chemotherapeutic and immunomodulating properties within a single nanoparticle formulation. The addition of alendronate, a potent amino-bisphosphonate, enhances PLAD's immunomodulatory effects by targeting macrophage and γδ T cell function [33]. It is worth noting that no experimental studies testing liposomal anthracyclines in human derived pancreatic cancer models have been published to the best of our knowledge. By leveraging ketotifen's ability to remodel the PDAC TME, this study aimed to determine if these nanomedicine formulations can achieve improved delivery and better therapeutic outcomes in murine models.

## 2 Results

### 2.1 Ketotifen is devoid of tumor inhibitory activity and has no significant effect on liposomal drug uptake in vitro

**Fig. 1:**
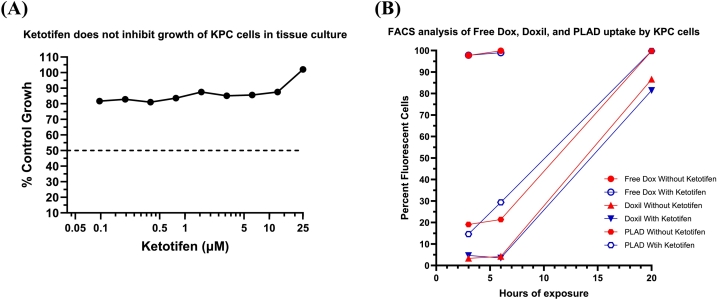
**(A)***In Vitro* Cytotoxicity test of Ketotifen on KPC cells after 72 h continuous exposure. **(B)** Flow Cytometry Analysis of Free Doxorubicin (DOX), Doxil or PLAD uptake with or without ketotifen treatment. Due to significant cytotoxicity, DOX analysis could not be performed at the 20 h time point for the Free Dox groups.

We first investigated the effect of ketotifen on KPC cell proliferation *in vitro*. We found that ketotifen showed no cytotoxic activity on KPC cells up to at least 25 μM after 72 h exposure (Fig. 1A). In addition, when co-incubated with Doxil or PLAD at 10 μM for 3 h, ketotifen did not alter either liposomal or free doxorubicin uptake, as indicated by FACS analysis of doxorubicin fluorescence-labelled cells after 3, 6, and 20 h incubation (Figs. 1B**, Supplementary Fig. S1).** As noted in the past, PLAD was taken up more avidly than Doxil by tumor cells [24].

### 2.2 Ketotifen enhances intratumoral liposome accumulation in implanted KPC tumors

**Fig. 2:**
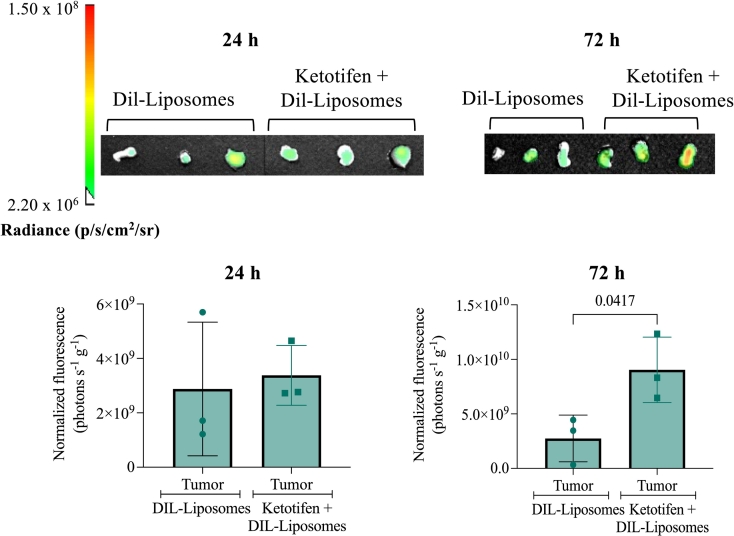
Intratumoral uptake of DIL-loaded liposomes. Mice bearing KPC tumors were treated for three days with ketotifen administered via intraperitoneal injection (i.p.) at a dose of 10 mg/kg once the tumors reached an average size of 150–200 mm^3^. Following the 3-day treatment, Dil-loaded liposomes were administered via intravenous (i.v.) injection. Tumors were excised and imaged *ex vivo* at 24 h and 72 h post-injection (*n* = 3 mice per timepoint) to assess Dil fluorescence (Em 610/ Ex 535). Fluorescence intensities were normalized to tumor weight. Data are presented as mean ± SEM. Statistical significance was determined using an unpaired *t*-test (two-tailed).

Next, we sought to determine whether ketotifen enhances both the uptake and retention of liposomes within tumors, while also assessing potential effects on liposome distribution in non-target organs, to provide insights into its impact on overall drug biodistribution. Mice bearing KPC tumors were pretreated with ketotifen (10 mg/kg daily for three days) prior to the administration of Dil-loaded, drug-free liposomes with lipid composition and size (80–100 nm) identical to Doxil and PLAD. Fluorescence imaging was performed at 24 and 72 h post-administration to evaluate liposome accumulation within tumors, as well as their distribution to major organs. At 24 h post-administration, there was no significant difference in liposome uptake between the groups treated with Dil-loaded liposomes alone and those pretreated with ketotifen (Fig. 2). However, at 72 h, a significant difference was observed, with the ketotifen-treated group showing enhanced liposome retention in tumors (Fig. 2**,** Supplementary Fig. S2). These findings indicate that ketotifen improves liposome accumulation within tumors over time, consistent with the combined effect of the enhanced permeability and retention (EPR) phenomenon of PEGylated long-circulating liposomes [34] and ketotifen-induced tumor retention.

Regarding non-tumor tissues, at 24 h post-administration, both groups (control Dil-loaded liposomes and ketotifen + Dil-loaded liposomes) showed high uptake in the liver and spleen, with minor accumulation detected in the lungs. No liposomes were detected in the kidneys or heart. At 72 h, Dil-loaded liposomes were largely cleared from most organs in both groups, except for liver and spleen in the ketotifen-treated mice, where liposomes remained detectable (Supplementary Fig. S2).

### 2.3 Ketotifen enhances the anti-tumor efficacy and improves survival of low dose Doxil and PLAD

**Fig. 3:**
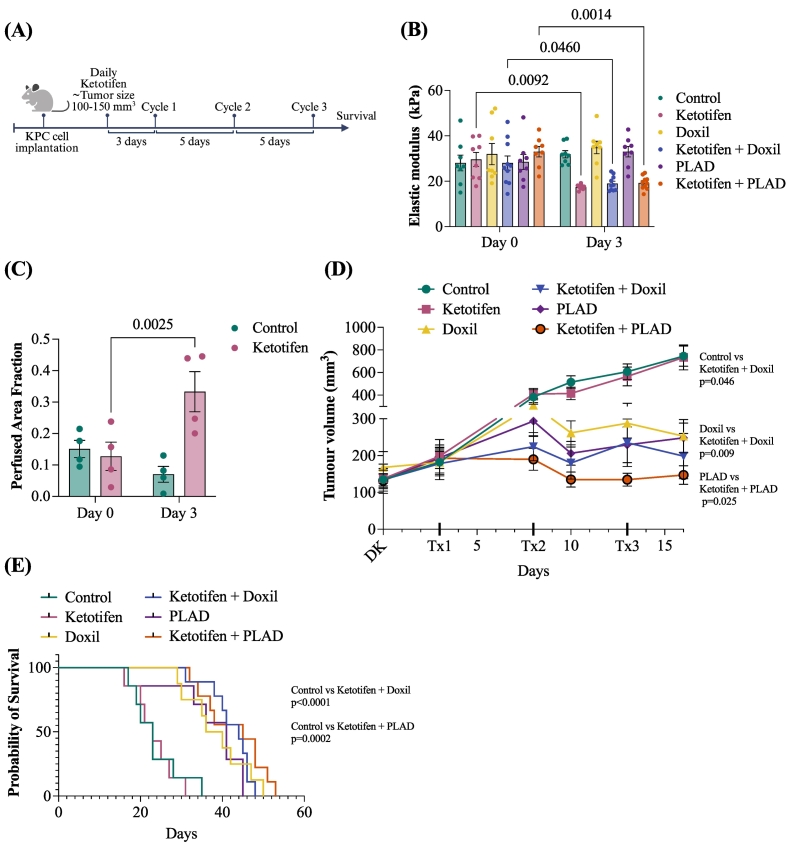
**(A)** Study treatment outline. Daily ketotifen was administered via intraperitoneal injection (i.p.) at a dose of 10 mg/kg once the tumors reached an average size of 100–150 mm^3^. Three cycles of PLAD or Doxil at a dose of 2.5 mg/kg were administered via intravenous injection (i.v.) with a 5-day interval between cycles. **(B)** Elastic modulus in kPa, showing the difference before and after ketotifen treatment. Data are presented as mean ± SEM (*n* = 8 per group). Statistical analysis was performed using a two-way ANOVA (Sidak's multiple comparisons test). (**C**) Perfused area fraction measured with CEUS. Data represent the mean ± SEM (*n* = 4 per group). Statistical analysis was performed using a two-way ANOVA (Sidak's multiple comparisons test). **(D)** Growth curves treated as indicated until day 16 (*n* = 7–10 mice per group). Data are presented as mean ± SEM. Statistical analysis were performed using a paired *t*-test (two-tailed). DK: Daily ketotifen **(E)** Kaplan-Meier curves for each treatment group. Survival outcomes were assessed based on either time to death following treatment initiation or time taken for the tumors to reach excessive burden (defined as 1200 mm^3^). Statistical significance was determined using the log-rank (Mantel-Cox) test. Hazard ratios (HR) and 95 % confidence intervals were also calculated.

Following the analysis of ketotifen's effect on liposome accumulation, we next examined whether its tumor microenvironment-modulating properties could enhance the therapeutic efficacy of liposomal nanomedicines (Doxil or PLAD) and improve survival in the KPC pancreatic tumor model. Mice bearing KPC tumors were treated with daily ketotifen (10 mg/kg) once tumors reached an average size of 100–150 mm^3^. Three days after initiating ketotifen, the first cycle of Doxil or PLAD (2.5 mg/kg) was administered via intravenous injection, with subsequent cycles given every five days for a total of three cycles [18]. Overall survival was assessed after the final cycle (Fig. 3A). Tumor stiffness, assessed via elastic modulus measurements using Shear Wave Elastography (SWE), showed a reduction from 40 kPa at baseline to approximately 20 kPa by day 3 in the treated groups (Fig. 3B**,** Supplementary Fig. S3A). Tumor blood volume (i.e., perfusion), assessed in control and ketotifen treated groups via Contrast Enhanced Ultrasound (CEUS), showed a significant increase in perfusion (Fig. 3C**,** Supplementary Fig. S3A). In the therapeutic assay, Doxil and PLAD monotherapies significantly inhibited tumor growth starting on day 10 after the second treatment cycle (*p* = 0.003 & *p* = 0.005 respectively). The addition of ketotifen enhanced significantly the efficacy of both therapies. By day 16, a significant difference was observed between Doxil vs. ketotifen + Doxil (*p* = 0.009) and PLAD vs. ketotifen + PLAD (*p* = 0.025) indicating that ketotifen further improved Doxil's and PLAD's ability to supress tumor growth (Fig. 3D). For the overall survival assessment, mice were monitored until tumors reached 1200 mm^3^. Treatment with Doxil or PLAD alone significantly prolonged survival compared to controls (Doxil: median 38 vs. 23 days, χ^2^ = 10.99, *p* = 0.0009; Hazard Ratio (HR) = 0.25, 95 % CI-0.07-0.93; PLAD: median = 41 vs. 23 days, χ^2^ = 7.11, *p* = 0.0077; HR = 0.31, 95 % CI = 0.09–1.08). Combination treatment with ketotifen and either Doxil or PLAD modestly extended median survival compared to the respective monotherapies (Fig. 3E). Specifically, median survival increased from 38 to 44 days for Doxil vs. ketotifen + Doxil (log-rank χ^2^ = 0.08, *p* = 0.78; HR = 0.88, 95 % CI = 0.34–2.30), and from 41 to 45 days for PLAD vs. ketotifen + PLAD (χ^2^ = 2.33, *p* = 0.13; HR = 0.52, 95 % CI = 0.17–1.52). Although these differences were not statistically significant, both trends were consistent with the improved drug accumulation and perfusion observed upon ketotifen treatment. Importantly, when compared to controls both combination groups showed a significant survival advantage. The ketotifen + Doxil group achieved a median survival of 44 days vs. 23 days for controls (χ^2^ = 15.94, *p* < 0.0001; HR = 0.21, 95 % CI = 0.05–0.85), while the ketotifen + PLAD group reached 45 vs. 23 days (χ^2^ = 14.05, *p* = 0.0002; HR = 0.22, 95 % CI = 0.05–0.88). Individual tumor growth curves up to day 55 are shown in Supplementary Fig. S3B-C. To further quantify overall tumor burden across treatment groups, the area under the curve (AUC) for each mouse was calculated from tumor growth data (Supplementary Fig. S3D) as previously described [35]. Consistent with the longitudinal measurements, AUC analysis confirmed significant tumor growth inhibition in all treatment groups compared to control.

Mouse body weight was monitored throughout the study as an indicator of toxicity. In the PLAD and ketotifen + PLAD treatment groups, 2 out of 9 mice exhibited significant toxicity, defined as >20 % body weight loss, after the third treatment cycle (Supplementary Fig. S4A-B). These mice were removed from the study between days 15 and 20.

**Fig. 4:**
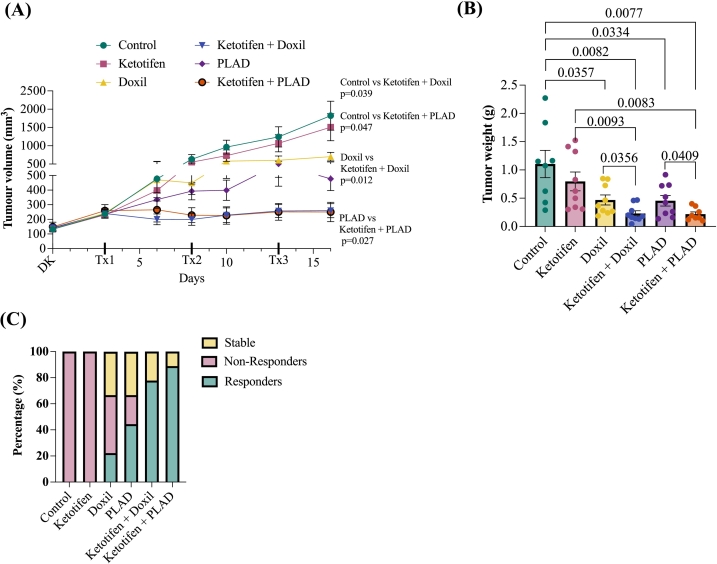
**(A)** Tumor growth curves treated as indicated until day 16 (*n* = 8–9 mice per treatment group). Data are presented as the mean ± SEM. Statistical analysis was performed using a paired *t*-test (two-tailed). DK: daily ketotifen **(B)** Tumor weight in g after excision on day 16 (n = 8–9 per group). Data are presented as mean ± SEM. Statistical analysis were performed by comparing means using an unpaired t-test with Welch's correction. (**C**) Response distribution per treatment group according to RECIST criteria, defining responders (≥30 % decrease in the major tumor diameter), stable disease (<30 % decreaase or < 20 % increase), and non-responders (≥20 % increase) at study end point.

To more accurately assess the potential of ketotifen in enhancing the anti-tumor efficacy of Doxil and PLAD, mice (both female and male to 1:1 ratio) bearing KPC tumors were treated with a reduced dose for both treatments (i.e., 1.75 mg/kg). The same treatment schedule was used as previously described (Fig. 3A). However, instead of monitoring survival, mice were sacrificed three days after the third and final treatment cycle, and tumors along with serum samples were collected for analysis. Doxil and PLAD monotherapies exhibited an anti-tumor effect as compared to control despite the low dose without any signs of toxicity. Interestingly, PLAD demonstrated superior efficacy compared to Doxil monotherapy (Fig. 4A). The addition of ketotifen to Doxil or PLAD further significantly enhanced tumor growth inhibition (Fig. 4A and Supplementary Fig. S5A). Tumors treated with ketotifen followed by Doxil and PLAD were also significantly smaller in weight (*ex-vivo*) compared to the monotherapy groups (Fig. 4B and Supplementary Fig. S5B). Consistent with these findings, analysis of tumor growth AUC reflected a similar trend toward reduced overall tumor burden in the ketotifen combination groups compared to the corresponding monotherapies (Supplementary Fig. S5C). In addition, treatment response distribution analysis (Fig. 4C) further highlighted the therapeutic benefit of ketotifen. Based on RECIST criteria adapted from preclinical models [36,37], mice were classified as responders (≥ 30 % reduction in the major tumor diameter), stable disease (<30 % decrease or < 20 % increase), or non-responders (≥ 20 % increase). In the monotherapy groups, treatment outcomes were heterogenous, with a mix of responders, non-responders, and cases of stable disease. In contrast, all animals in the ketotifen combination groups exhibited objective tumor responses, with the proportion of responders increasing to 78 % and 89 % for the ketotifen + Doxil and ketotifen + PLAD groups, respectively compared to 20 % and 42 % responders for the groups treated only with Doxil or PLAD. Minimal and nonsignificant changes in body weight were observed in the treatment groups under this treatment regime (Supplementary Fig. S6).

### 2.4 Impact of treatment on tumor and serum cytokine levels

**Fig. 5:**
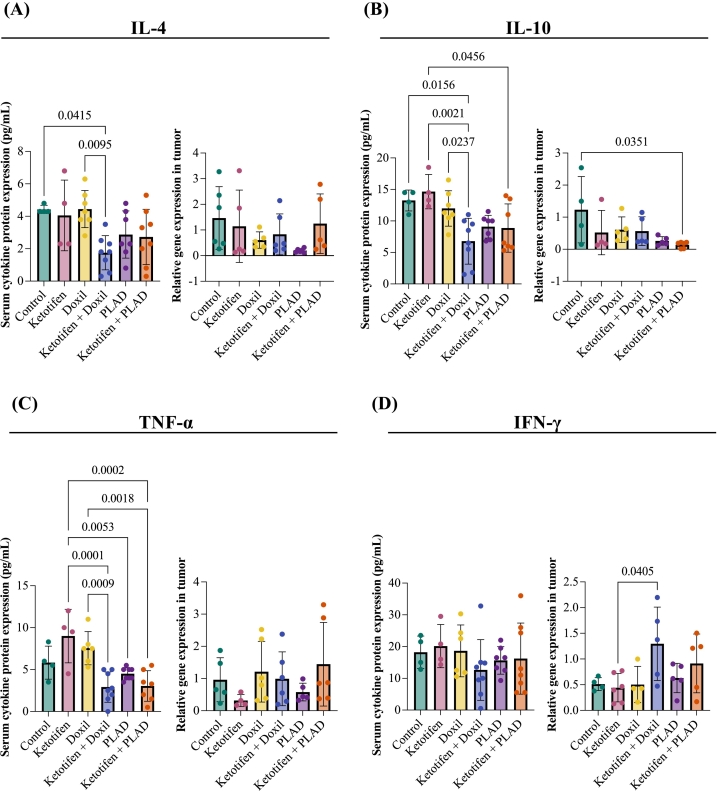
**A-D:** Concentrations of serum cytokines analyzed by Bio-Plex multiplex cytokine assay and mRNA gene expression levels in tumor tissue lysate samples at 72 after the third and final treatment cycle. Data are presented as mean ± SEM. Statistical analysis were performed by ordinary one-way ANOVA using Tukey's multiple comparisons test.

To further investigate the effects of ketotifen on PDAC treatment, we performed a multiplex assay and RT-qPCR to analyze serum cytokine levels and tumor tissue, respectively. Cytokine expression in the TME was evaluated 72 h after the third and final treatment cycle using RT-qPCR analysis of tumor tissue, while systemic cytokine levels were measured in serum using a multiplex assay. Among the treatment groups, a significant reduction in serum IL-10 levels was observed, while IL4 and TNF-α showed a non-significant decreasing trend (Fig. 5A-C). In tumor tissue, mRNA expression of IL-4 and TNF-α remained largely unchanged across groups. However, IL-10 expression was significantly reduced in the ketotifen + PLAD group compared to control.

Interestingly, tumor tissue analysis revealed a modest increase in IFN-γ expression in the ketotifen + Doxil group compared to ketotifen alone (Fig. 5D). No other significant differences were detected in the serum cytokines analyzed (Supplementary Fig. S7). These cytokine changes suggest that treatment may influence the immune landscape of the TME, potentially contributing to the observed therapeutic effects, but failed to provide a valid marker significantly correlated with ketotifen treatment or chemotherapy type.

### 2.5 Multivariate analysis of biomarkers associated with treatment response

**Fig. 6:**
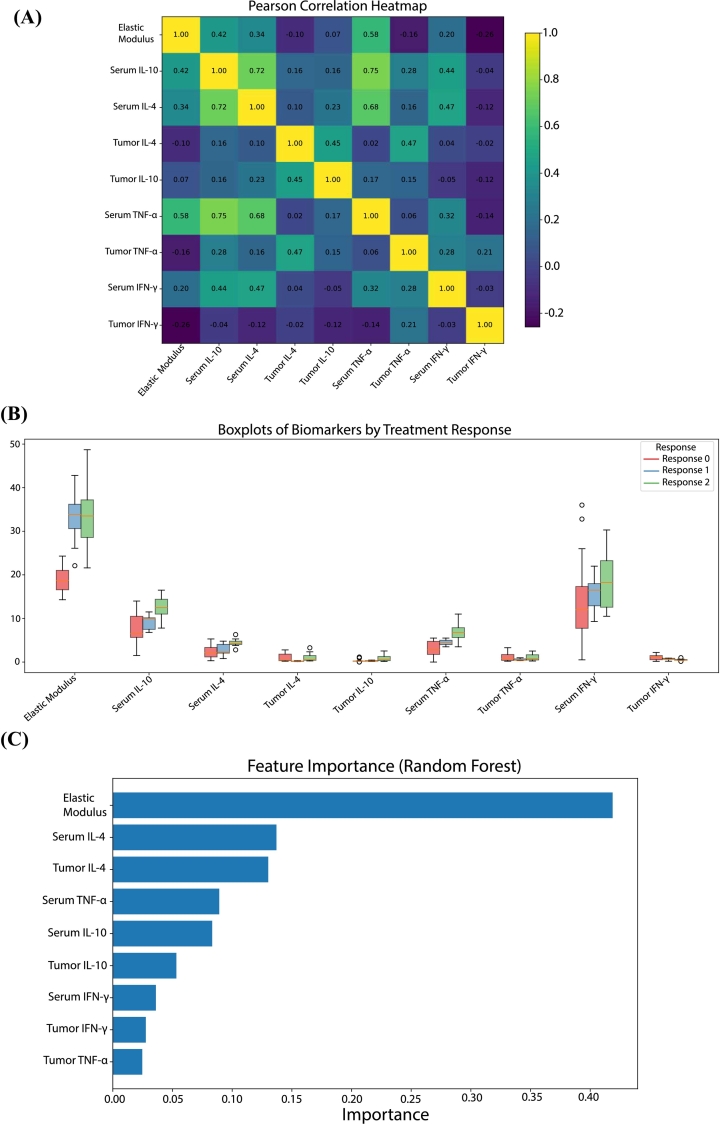
*(A)* Pearson correlation heatmap of elastic modulus and cytokine expression levels in tumor-bearing mice. Strong positive correlations were observed between certain immune markers (e.g., IL-4 and IL-10), as well as between Multiplex TNF and tumor stiffness (Elastic Modulus), indicating potential interdependencies between immune and mechanical components of the TME. (B) Boxplots illustrating the distribution of key biomarkers and elastic modulus across treatment response categories (non-responders, partial responders, full responders). Differences in Elastic Modulus, Multiplex IL-10, and IL-4 levels were evident, with higher values associated with poor treatment response. Data suggest a mechanistic role for these parameters in predicting therapeutic efficacy. (C) Feature importance scores derived from a Random Forest classifier trained to distinguish between treatment response groups using mechanical and immunological biomarkers. Multiplex IL-10, Multiplex TNF, and Elastic Modulus emerged as the most predictive features.

To identify biomarkers associated with individual treatment response, we conducted an integrative analysis combining statistical visualization and machine learning-based feature selection. Pearson correlation analysis revealed strong positive associations between several cytokines, including IL-10 and IL-4 (*r* = 0.73), and between TNF-α and Elastic Modulus (*r* = 0.63), highlighting, potential links between immune signalling and mechanical properties of the TME (Fig. 6A). Boxplot comparisons (Fig. 6B) across response groups revealed distinct patterns: elastic modulus and IL-10 (protein, multiplex) levels were elevated in non-responders, whereas relative mRNA expression of cytokines such as *Il4* and *Ifnγ* (by RT-qPCR) tended to be lower in these mice. These differences point toward immune suppression and increased tumor stiffness in poorly responding tumors. To assess the robustness and generalizability of the Random Forest classifier, a 5-fold stratified cross-validation was implemented. The model achieved a mean accuracy = 0.82, macro F1 = 0.73, and macro ROC–AUC = 0.92, indicating strong discrimination between response categories (**Supplementary Fig. S8**). The corresponding Precision–Recall (PR) analysis yielded a macro average precision (AP) of 0.84, confirming stable predictive performance (**Supplementary Fig. S8**). The confusion matrix (**Supplementary Fig. S8**) revealed limited overlap between stable and non-responder groups, consistent with their partially shared cytokine profiles [23]. The most influential variables included IL-10, TNF-α (analyzed by multiplex), and, importantly, elastic modulus (Fig. 6C). These results support the utility of mechanical and immunological biomarkers in stratifying treatment response.

## 3 Discussion

In this study, we investigated the role of ketotifen in modulating the TME and enhancing the efficacy of nanomedicines (Doxil and PLAD), in a preclinical KPC pancreatic tumor model. Our findings demonstrate that ketotifen significantly reduces tumor stiffness, as evidenced by the reduction in elastic modulus following a three-day treatment, consistent with previous observations in sarcoma models [15,18,22]. These results suggest that the beneficial effects of ketotifen are not restricted to a specific tumor type but represent a broader strategy applicable to multiple solid malignancies. While Doxil and PLAD monotherapies effectively inhibited tumor growth, and prolonged survival, ketotifen was introduced to further enhance their anti-tumor efficacy by normalizing the TME. Importantly, we found that ketotifen can improve intratumoral delivery and retention of nanomedicines even at low doses. As plasma levels of liposomal drugs remain unchanged with and without ketotifen pretreatment [18] and *in vitro* studies confirm lack of direct cytotoxic effect (Fig. 1 and **Supplementary Fig. S1**), the observed enhancement in tumor delivery can be attributed to improved tumor transport properties. Collectively, these observations support the conclusion that ketotifen enhances nanomedicine accumulation by reducing tumor stiffness and improving vascular perfusion. Mechanistically, ketotifen functions as a mast-cell stabilizer that prevents degranulation and reduces the release of profibrotic and vasoactive mediators, such as TGF-β, thereby limiting CAF activation and ECM deposition. These processes lead to vascular decompression and restore perfusion, consistent with the mechanical softening and enhanced liposome accumulation observed here [15]. At 72 h post-injection, *ex vivo* fluorescence imaging revealed increased liposome-associated signal in tumors of ketotifen-treated mice, consistent with improved perfusion enabling deeper drug penetration. Fluorescence in the liver and spleen, key organs of the mononuclear phagocyte system (MPS), remained within expected ranges for long-circulating liposomes and showed no significant difference with ketotifen pretreatment, aligning with our previous finding that mechanotherapeutic TME modulation selectively alters tumor stiffness without affecting healthy tissues [38]. While *ex vivo* fluorescence imaging provided valuable comparative information on intratumoral retention it does not directly quantify absolute drug levels. To minimize variability, all imaging parameters were standardized and signals normalized to tissue mass, and importantly, the enhancement induced by ketotifen remained significant.

Although, survival improvements in the ketotifen-treated groups did not reach statistical significance at the standard dosing regimen, the therapeutic benefit of ketotifen became more evident when Doxil and PLAD doses were reduced to minimize toxicity. Low-dose monotherapies exhibited weaker tumor control but their combinations with ketotifen preserved strong anti-tumor activity. This highlights ketotifen's clinical value in maintaining treatment efficacy under dosing constraints aimed at improving safety. Treatment-response distribution analysis (using RECIST criteria) further supported this conclusion. Monotherapy groups showed heterogeneous outcomes, including responders, non-responders, and stable disease cases, whereas all ketotifen combination-treated animals exhibited objective responses, with responder proportions, increasing to 78 % and 89 % for the ketotifen + Doxil and ketotifen + PLAD groups, respectively, indicating a more consistent and robust therapeutic effect across animals.

To explore whether immune modulation contributes to ketotifen-associated improvements, we evaluated immune-related cytokines in serum and tumor tissue. Reduced IL-4 and IL-10 levels in ketotifen-treated groups suggest a shift away from an immunosuppressive M2-like environment, while modest increases in IFN-γ indicate possible enhancement of local inflammatory activation [[39], [40], [41]]. PLAD's full immunotherapeutic potential cannot be captured in mice due to the absence of Vγ9Vδ2 T cells, its primary target population in humans, which may partially explain its comparable activity to Doxil in this model [42].

Together, our data support a mechanistic sequence wherein ketotifen remodels the PDAC stroma, improves perfusion and drug distribution, leading to improved tumor response, rather than a simple additive pharmacologic effect. This study has, however, several limitations: First, therapeutic studies were performed in subcutaneous KPC-tumors to enable repeated SWE and CEUS imaging, whereas orthotopic models might better recapitulate pancreatic anatomy but are technically challenging for ultrasound imaging. There is, however, evidence that ectopic KPC tumors still resemble the stroma composition and immune characteristics of orthotopic tumors [20,26,28,29]. Second, fluorescence imaging provides relative rather than quantitative measurements of drug content and lacks spatial resolution to distinguish vascular pooling from deep interstitial penetration; analytical methods such as high-performance liquid chromatography (HPLC) or LC-MS could provide quantitative assessment of intratumoral drug concentrations. Third, the immune analysis focused on cytokine expression only, without characterizing immune cell infiltration or activation state, as the primary objective of this study was to evaluate therapeutic response rather than conduct detailed immune profiling. Fourth, toxicological evaluation was limited, as reduced-dose regimens were well tolerated and did not warrant additional biomarkers. In this context, comprehensive safety studies will be required for clinical translation. Finally, all experiments were performed in a single KPC-derived tumor model, and future studies incorporating multiple orthotopic and patient-derived models would be necessary to validate broader applicability across PDAC subtypes.

Overall, this work highlights the potential of ketotifen as a clinically translatable agent to remodel the TME, enhance nanomedicine delivery, and strengthen anti-tumor efficacy in pancreatic cancer. Given ketotifen's established safety profile and regulatory approval in human [43], its repurposing as an adjuvant to nanoparticle-based therapies may offer a promising strategy to overcome drug-delivery barriers in PDAC.

## 4 Methods

### 4.1 Drugs

Ketotifen fumarate salt (K2628, Sigma) was dissolved in sterilized saline (0.9 % NaCl in dH_2_O, *w*/*v*). Doxil/Caelyx™ PEGylated liposomal doxorubicin hydrochloride (10-ml vials, Dox concentration: 2 mg/ml) was obtained from Baxter Corporation (Deerfield, Illinois). PLAD was prepared as previously described, except for an additional step of extrusion through 0.05 μm pore size polycarbonate membrane filters [31]. The characteristics of the batch used in this study are: 20-ml vial, Dox 1.9 mg/ml, phospholipid 21.9 μmoles/ml, alendronate 4.6 μmoles/ml, osmolarity = 361 mOsm/l, pH 6.6, average vesicle size 66 nm (polydispersity index 0.05). Free drug was analyzed after separation by centrifugation of a liposomes sample in a Vivaspin® 500 tube with 300 K MW cutoff (Sartorius, Gottingen, Germany). Free Dox was not detectable, free alendronate was 0.8 %.

### 4.2 Cell culture

Kras-transformed murine pancreatic adenocarcinoma (KPC) cells were purchased from CancerTools.org. Cells were grown in Dulbecco's Modified Eagle Medium supplemented with 10 % Fetal Bovine Serum (FBS), 1 % antibiotic/antimycotic, and incubated in a CO_2_ incubator at 37 °C.

### 4.3 Cytotoxicity assay

KPC cells were seeded in a 96-well cell culture plate at 10,000 cells per well. Cells were incubated at 37 °C under 5 % CO2 with different concentrations of Ketotifen for 72 h. Cell growth was measured calorimetrically by a methylene-blue based assay in an Elisa reader at 620 nm as previously described [44].

### 4.4 FACS analysis

The cultured KPC cells were seeded in 35 mm 6-well plates at a concentration of 5 × 10^5^ cells/well. Upon 24 h post-seeding, the media were replaced with fresh media or media containing 10 μM ketotifen, followed by the addition of 2 μM concentrations of Dox, Doxil, or PLAD, and incubated for different time points (3, 6, and 20 h). After incubation, the media were removed, and the cells were washed with PBS (2 ml × 2), followed by trypsin treatment to detach the cells and fixation with 2 % paraformaldehyde. The fixed cells were resuspended in 500 μl PBS and transferred to a BD FACS Canto II analyzer for flow cytometry (BD Biosciences, NJ).

### 4.5 Tumor model

Ectopic pancreatic tumor models were generated by subcutaneous implantation of 1.5 × 10^6^ KPC cells resuspended in 50 μl of PBS into 8–10-week-old C57BL/6 mice. All mice were maintained in specific pathogen-free conditions in the animal facilities of Cyprus Institute of Neurology and Genetics. All experiments were conducted in accordance with the animal welfare regulations and guidelines of the European Union (European Directive 2010/63/EE and Cyprus Legislation for the protection and welfare of animals, Laws 1994–2013) under a license acquired and approved (CY/EXP/PR.L03/2020) by the Cyprus Veterinary Services committee, the Cyprus national authority for monitoring the welfare of animals in research.

### 4.6 Treatment protocols

#### 4.6.1 Survival and therapeutic studies

Mice bearing KPC tumors (tumor size approximately 150-180 mm^3^) were treated daily with ketotifen administered via intraperitoneal injection at a dose of 10 mg/kg or equivalent volume of diluent (0.9 % NaCl saline). Day 0 was defined as the start of daily ketotifen treatment, when tumors reached the target size range. Three days later (Day +3), mice received the first cycle (Cycle 1) of PLAD or Doxil, administered either alone or in combination with ketotifen (2.5 mg/kg for survival study, 1.75 mg/kg for therapeutic study). Each treatment cycle was separated by a 5 day-interval (Cycle 2 on Day +8), Cycle 3 on Day +13). Tumor progression was monitored throughout and after the final treatment cycle, and survival outcomes were assessed based on either the time to death following treatment initiation or time taken for the tumors to reach excessive burden (defined as 1200 mm^3^). Planar dimensions (x.y) of tumor were monitored every 2–3 days using a digital caliper and tumor volume was estimated from the volume of an ellipsoid and assuming that the third dimension, z, is equal to the average of the other two.

#### 4.6.2 Biodistribution

C57BL/6 mice bearing KPC tumors, generated as described previously, were maintained on a low-fluorescence diet to minimize autofluorescence. Tumors were allowed to grow to an average size of 150–200 mm^3^. Prior to intravenous administration of Dil-loaded liposomes (200 μl, per mouse), the mice were pre-treated with ketotifen at a dose of 10 mg/kg. Following, 3 mice per group were sacrificed at 24 and 72 h post injection, and tumor, liver, kidneys, spleen, heart and lungs were excised and immediately imaged ex vivo at Em 610/ Ex 535 nm using an AMI HT Optical Imaging system (Spectral Instruments Imaging, US). All images were acquired under identical settings (exposure time, f/stop, binning, and field of view). Regions of interest (ROIs) were manually drawn around each tissue sample, and a background ROI of equal size was placed in a signal-free area for subtraction. The resulting background-corrected fluorescence (photons s^−1^) was normalized to tissue mass (g) and expressed as photons s^−1^ g^−1^.

#### 4.6.3 Multiplex Analysis

Serum cytokines were measured in duplicate, using a Bioplex Pro mouse cytokine multiplex kit (Bio-Rad, Gladesville, Australia, CAT # M60009RDPD), according to the manufacturer's instructions. Serum samples were assessed at 1:4 dilution. 50 μl of 1 x beads were added to each of the 96 wells, followed by two washes using 100 μl of wash buffer. 50 μl of standards, blank and serum samples were added to the appropriate wells and incubated at room temperature with shaking at 850 rpm, followed by three washes. 25 μl of detection antibody was added and incubated at room temperature at 850 rpm. After another 3 washes, 50 μl of streptavidin-PE was added and incubated at room temperature for 10 min at 850 rpm. A final three washes were performed, and the beads were resuspended in 125 μl of assay buffer. The plate was then read using a Multiplex Luminex xMAP. Cytokine concentrations (pg/ml) were calculated using analysis software based on standard curves per cytokine.

#### 4.6.4 Real-time PCR

Total RNA was extracted from tumor tissue using TRizol reagent (1 ml per 100 mg of tissue sample). RNA was reverse transcribed into cDNA using Superscript III Reverse Transcriptase (Invitrogen), and diluted cDNA (1:10) was used for quantitative real-time PCR (RT-qPCR) using *β*-actin as a reference gene. All primer sequences are listed in **Supplementary Table S1.** Quantification of relative gene expression was performed using ΔΔCT method.

#### 4.6.5 Shear Wave Elastography and Constrast Enhanced Ultrasound Imaging

Shear wave elastography (SWE) was used to measure elastic modulus. A Philips EPIQ Elite scanner with the eL18–4 linear array transducer was employed as previously described [18]. Mice were anesthetized with Avertin (tribromoethanol, 250 mg/kg, intraperitoneally) prepared as a 2.5 % (*w*/*v*) solution in tert-amyl alcohol and diluted in water. Body temperature was maintained at 37 °C using a heated imaging platform. CEUS was used to assess tumor associated vascular perfusion following the bolus administration of a microbubble contrast agents (Optison; GE Healthecare). The contrast agent was injected via retro-orbital vein under avertin anaesthesia. Tumor imaging was performed using the linear array transducer L12–5. Real-time power modulation imaging was initiated following a high mechanical index destruction pulse to rupture the microbubbles. Subsequent replenishment of contrast within the tumor vasculature was visualized and recorded, allowing assessment of vascular perfusion kinetics and peak contrast intensity.

### 4.7 Statistical and Bioinformatic analysis

Statistical analysis was performed using Prism (GraphPad; RRID: SCR_002798). Each test used in indicated in figure legends. To assess the molecular and mechanical predictors of treatment response, a comprehensive multivariate data analysis pipeline was implemented. Feature distributions across treatment response groups were evaluated using boxplots, while inter-variable relationships were explored through Pearson correlation analysis. A biplot was constructed to visualize both sample clustering and variable contributions to the principal components. Feature importance scores were extracted using a Random Forest classifier trained on the same dataset. Classification accuracy was validated using 5-fold cross-validation, and performance was visualized via confusion matrices. Statistical analysis and data visualization were performed using Python (scikit-learn, seaborn, matplotlib, pandas).

### 4.8 Model validation and performance

Model validation was performed using a 5-fold stratified cross-validation strategy to ensure balanced representation of all response classes. Performance metrics including accuracy, macro F1-score, and ROC–AUC were computed per fold and averaged. ROC and PR curves were generated to evaluate class-specific discrimination. The macro AUC = 0.92 and macro AP = 0.84 indicate high predictive performance. A confusion matrix summarizing out-of-fold (OOF) predictions was constructed to visualize classification performance [[45], [46], [47]] (Supplementary Fig. S8). Analyses were performed in Python using scikit-learn v1.3.2 and matplotlib v3.8.2.)

## CRediT authorship contribution statement

**Antonia Charalambous:** Writing – review & editing, Writing – original draft, Validation, Methodology, Formal analysis, Data curation. **Fotios Mpekris:** Writing – review & editing, Validation, Methodology. **Chrysovalantis Voutouri:** Writing – review & editing, Validation, Methodology, Formal analysis. **Constantina Neophytou:** Writing – review & editing, Validation, Methodology. **José Djamus:** Writing – review & editing, Validation, Methodology, Formal analysis. **Ajay Gupta:** Writing – review & editing, Validation, Methodology, Formal analysis. **Alberto Gabizon:** Writing – review & editing, Validation, Supervision, Resources, Methodology, Conceptualization. **Triantafyllos Stylianopoulos:** Writing – review & editing, Visualization, Validation, Supervision, Resources, Methodology, Funding acquisition, Conceptualization.

## Declaration of competing interest

The authors declare no potential conflicts of interest.

## Data Availability

Data will be made available on request.

## Appendix A Supplementary data

Supplementary material 1 Image 1

Supplementary material 2 Image 2

Supplementary material 3 Image 3

Supplementary material 4 Image 4

Supplementary material 5 Image 5

Supplementary material 6 Image 6

Supplementary material 7 Image 7

Supplementary material 8 Image 8

Supplementary material 9 Image 9
