# Supplementary material for: Enhancing nanomedicine efficacy in KPC pancreatic tumors through ketotifen-mediated tumor microenvironment remodeling
Source: J Control Release. 2026 Feb 10;390:114541. doi: 10.1016/j.jconrel.2025.114541 (PMC12888560; doi:10.1016/j.jconrel.2025.114541)

3 h Incubation

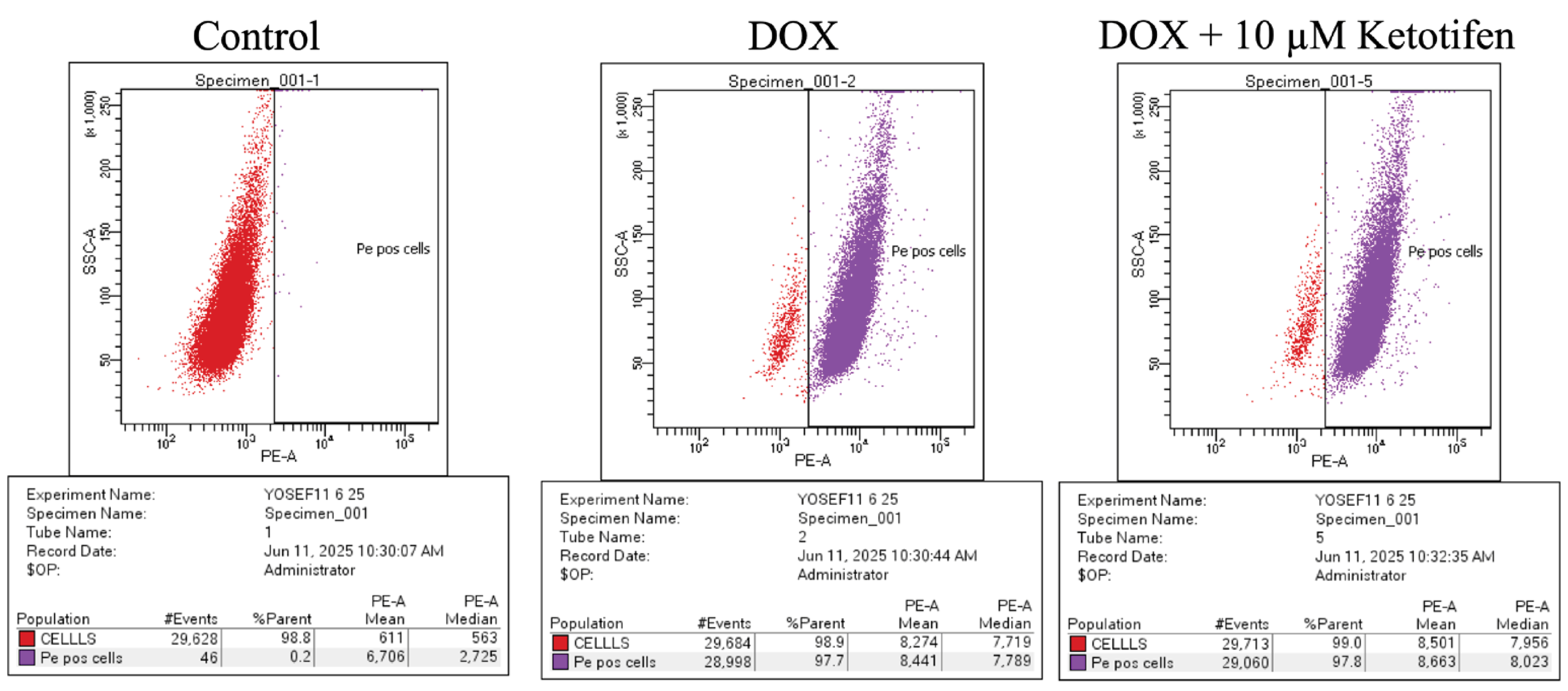

Conc. of DOX 2 μM

3 h Incubation

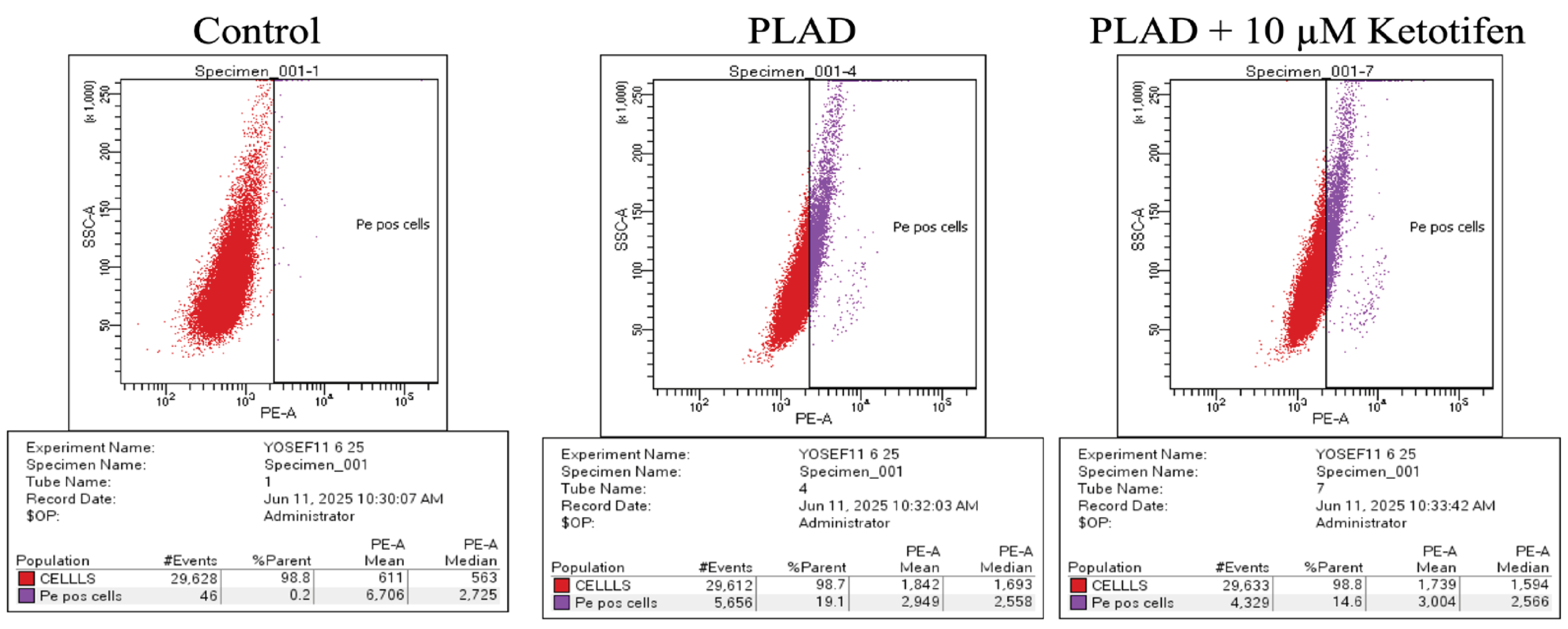

Conc. of PLAD 5 μM

3 h Incubation

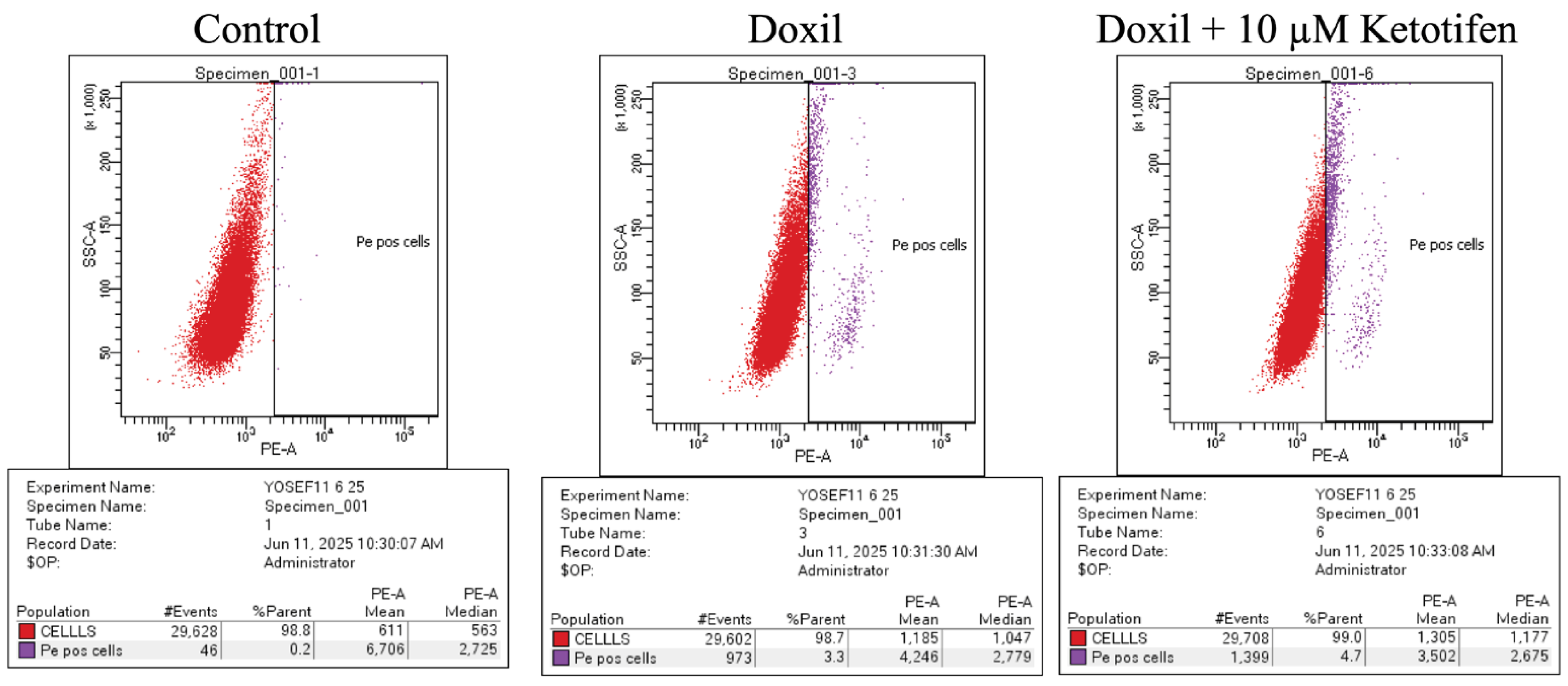

Conc. of Doxil 5 μM

6 h Incubation

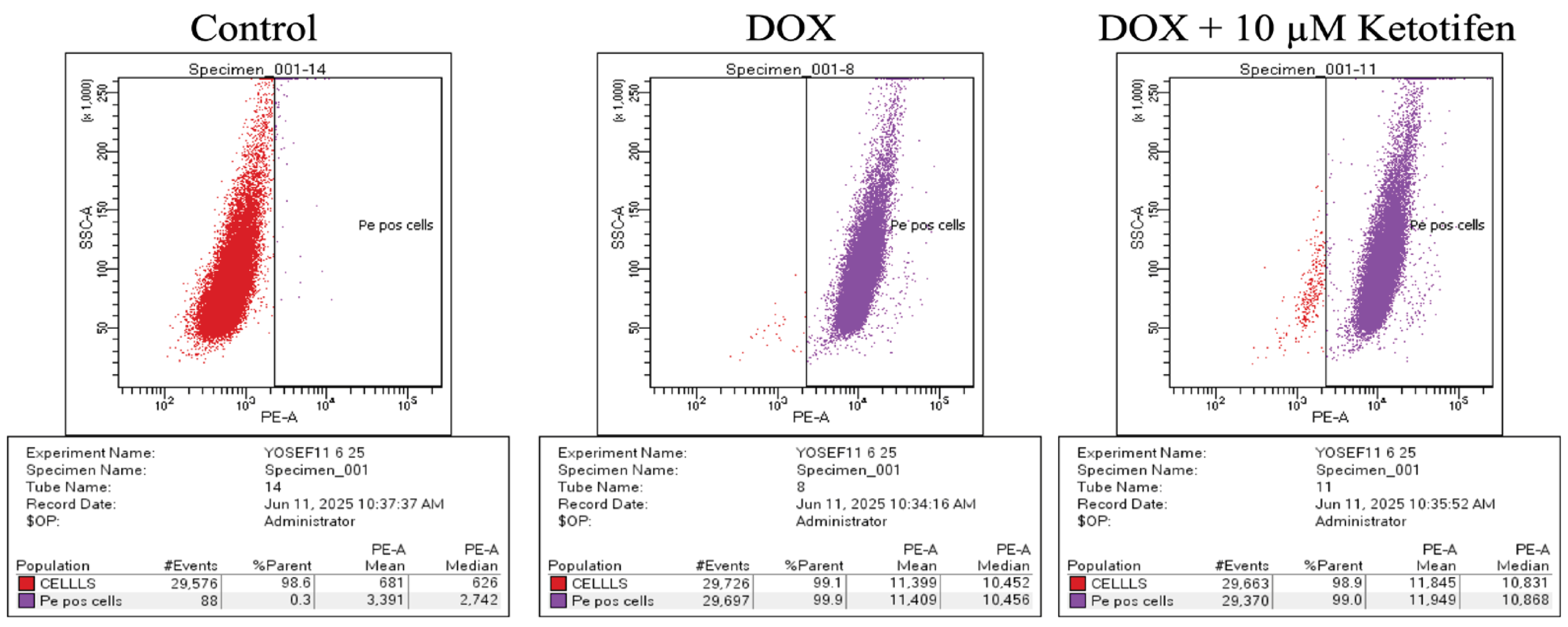

6 h Incubation

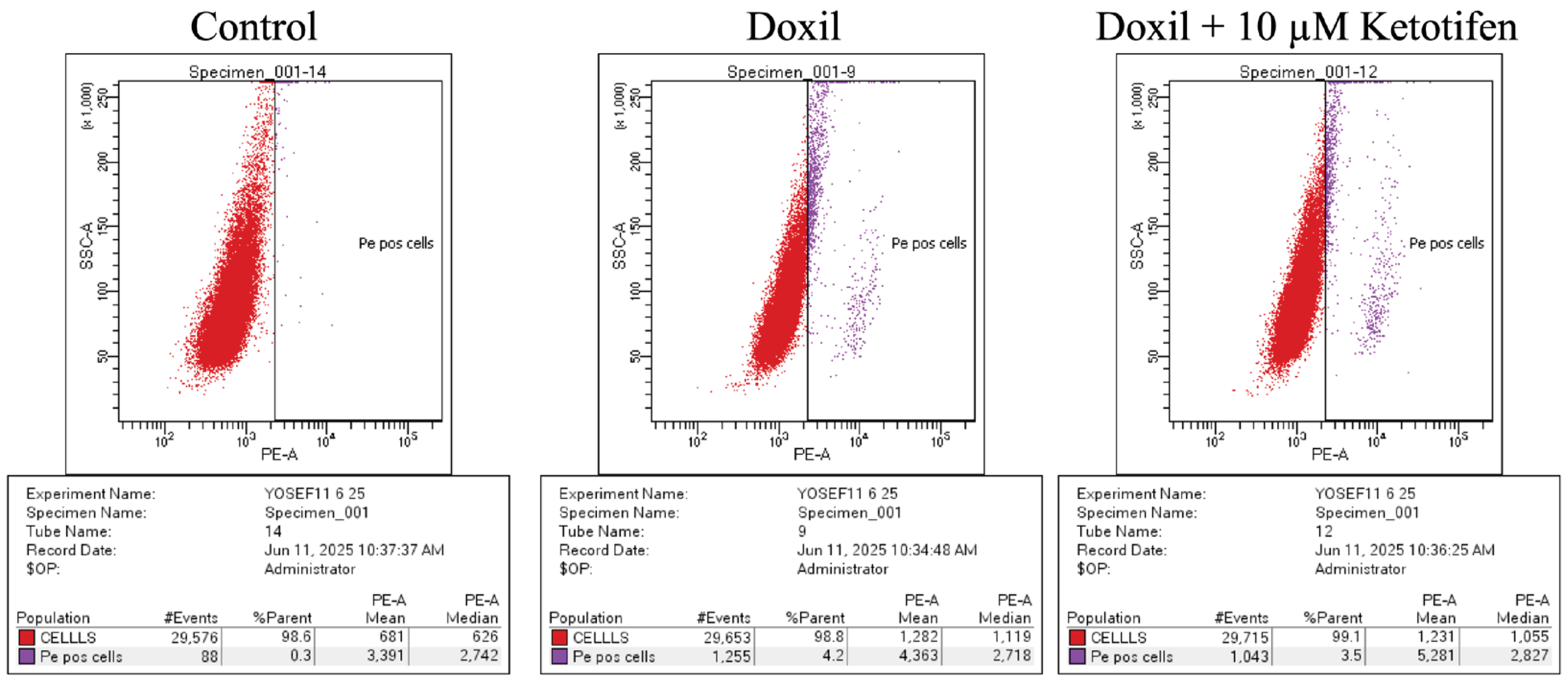

20 h Incubation

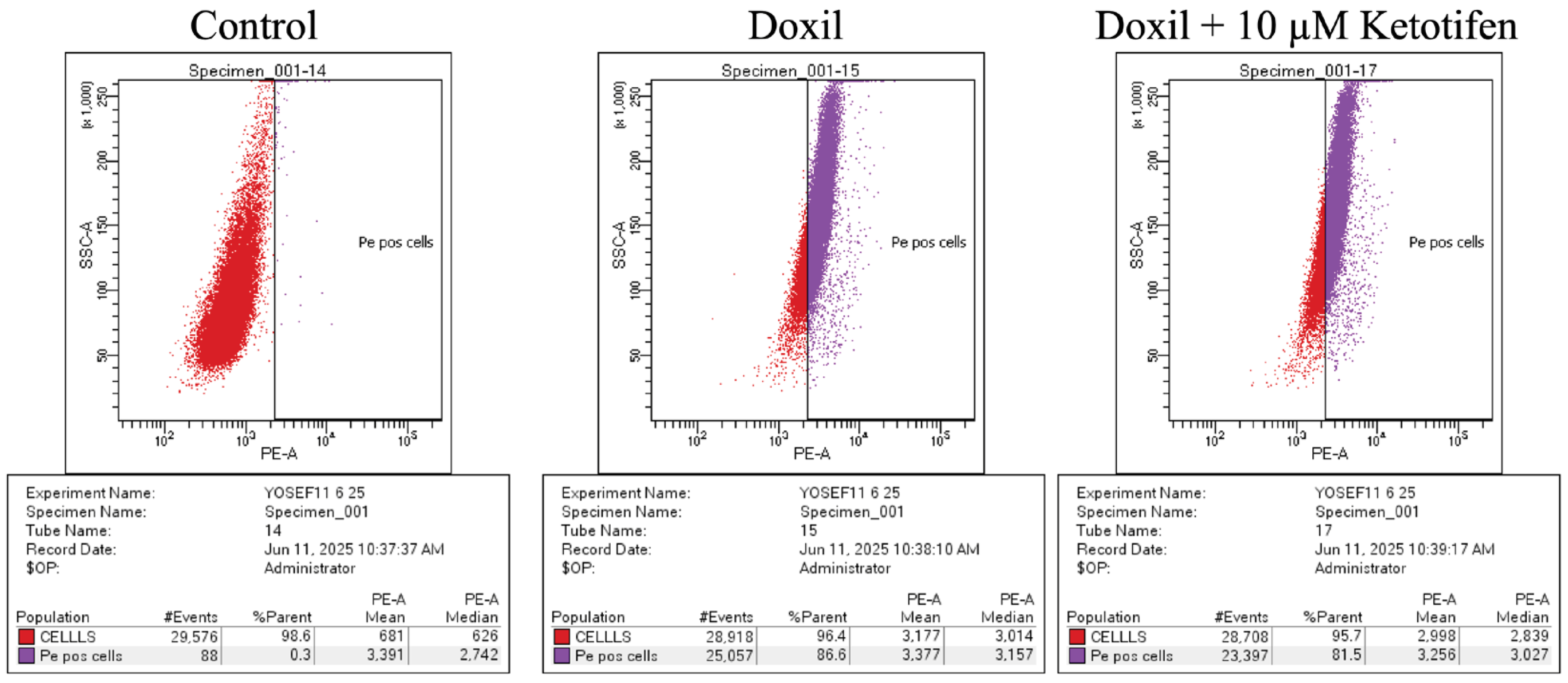

6 h Incubation

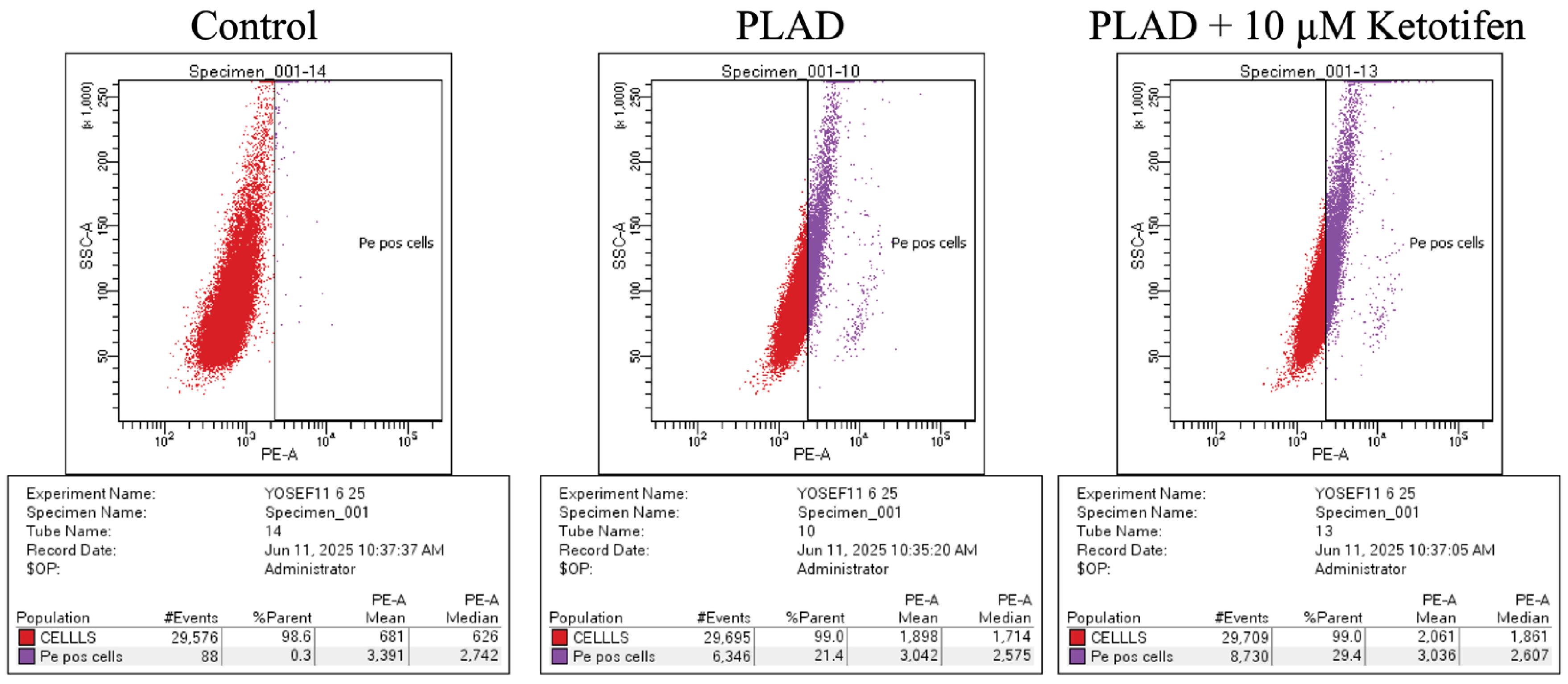

20 h Incubation

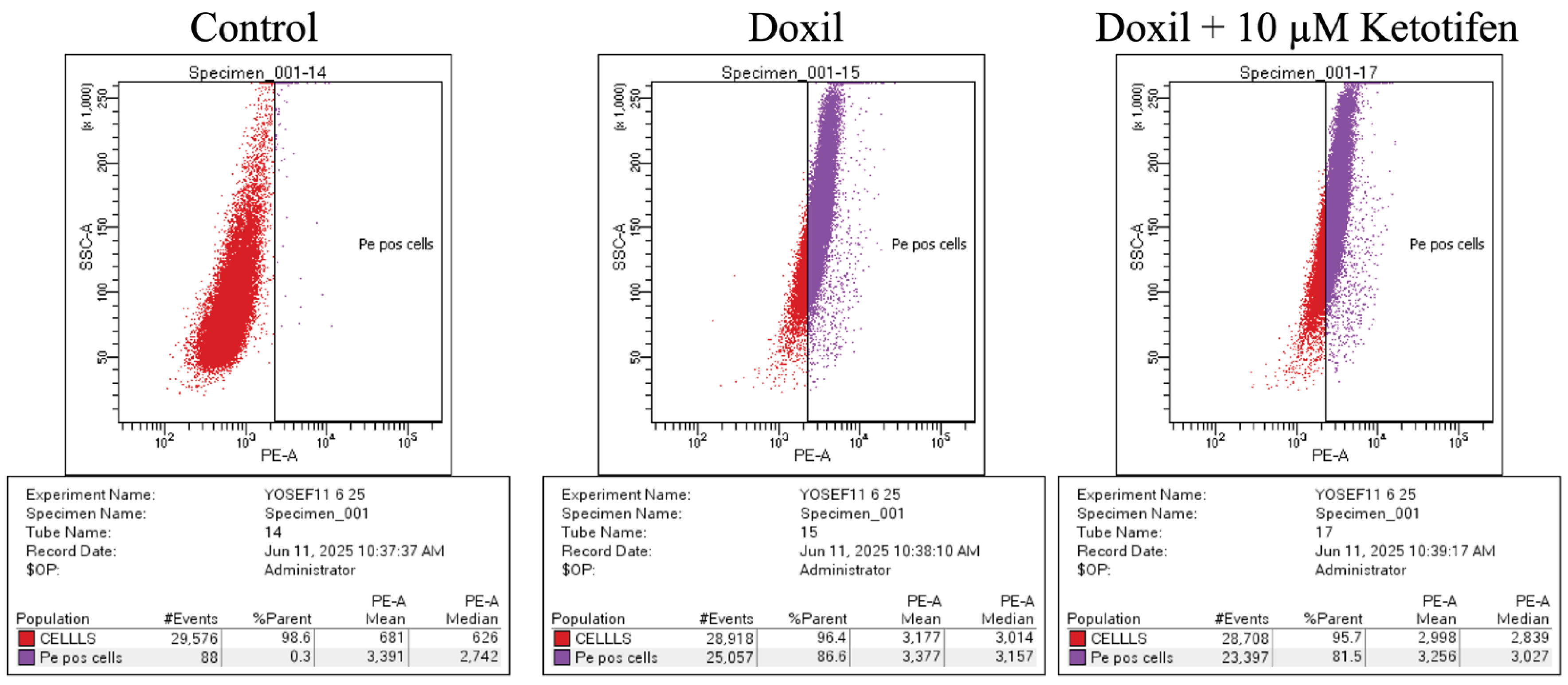

Supplement: Supplementary file 1 — Supplementary material 1 [file mmc1.pdf]
