# Supplementary figures and images for: Enhancing nanomedicine efficacy in KPC pancreatic tumors through ketotifen-mediated tumor microenvironment remodeling
Source: J Control Release. 2026 Feb 10;390:114541. doi: 10.1016/j.jconrel.2025.114541 (PMC12888560; doi:10.1016/j.jconrel.2025.114541)

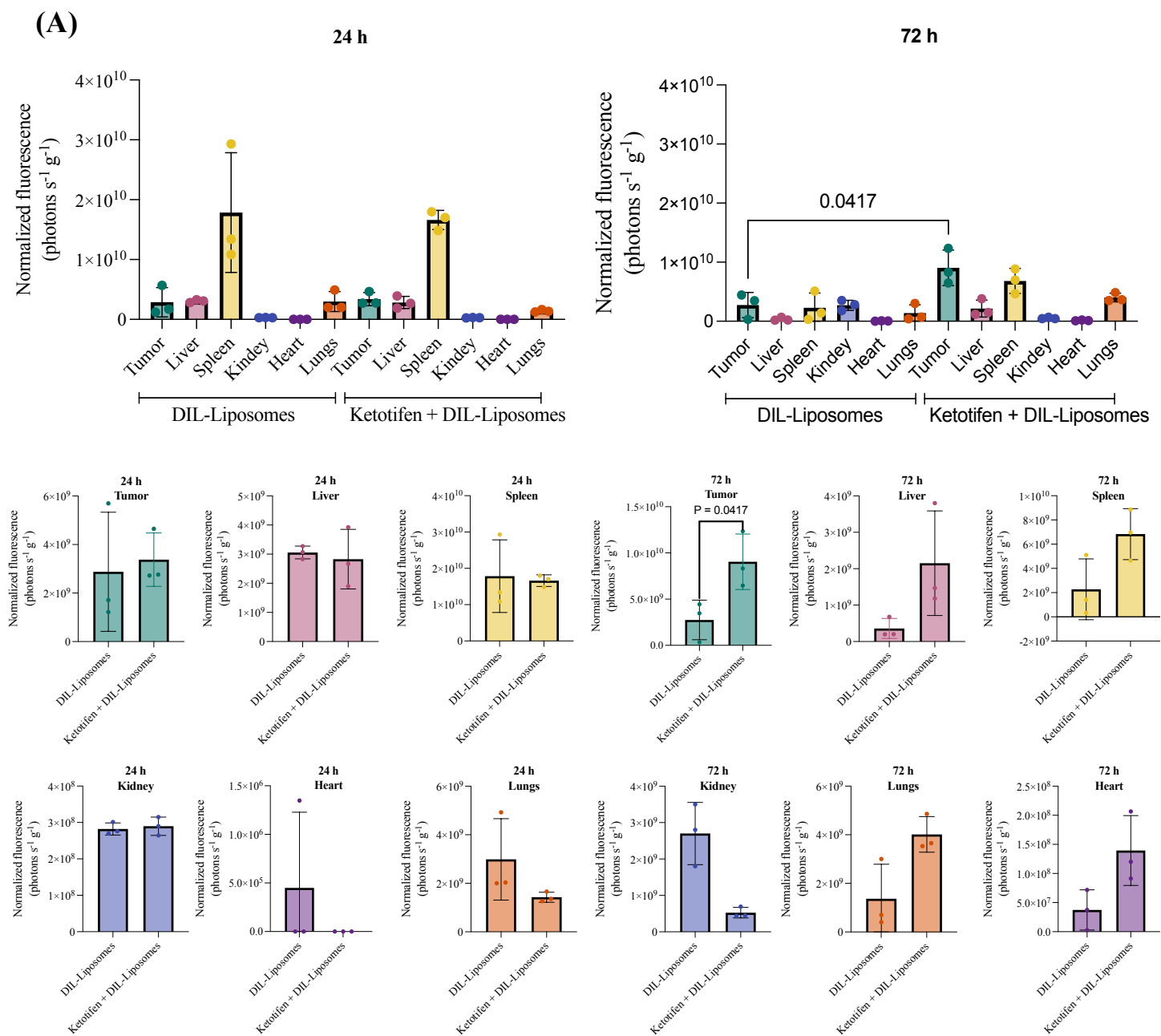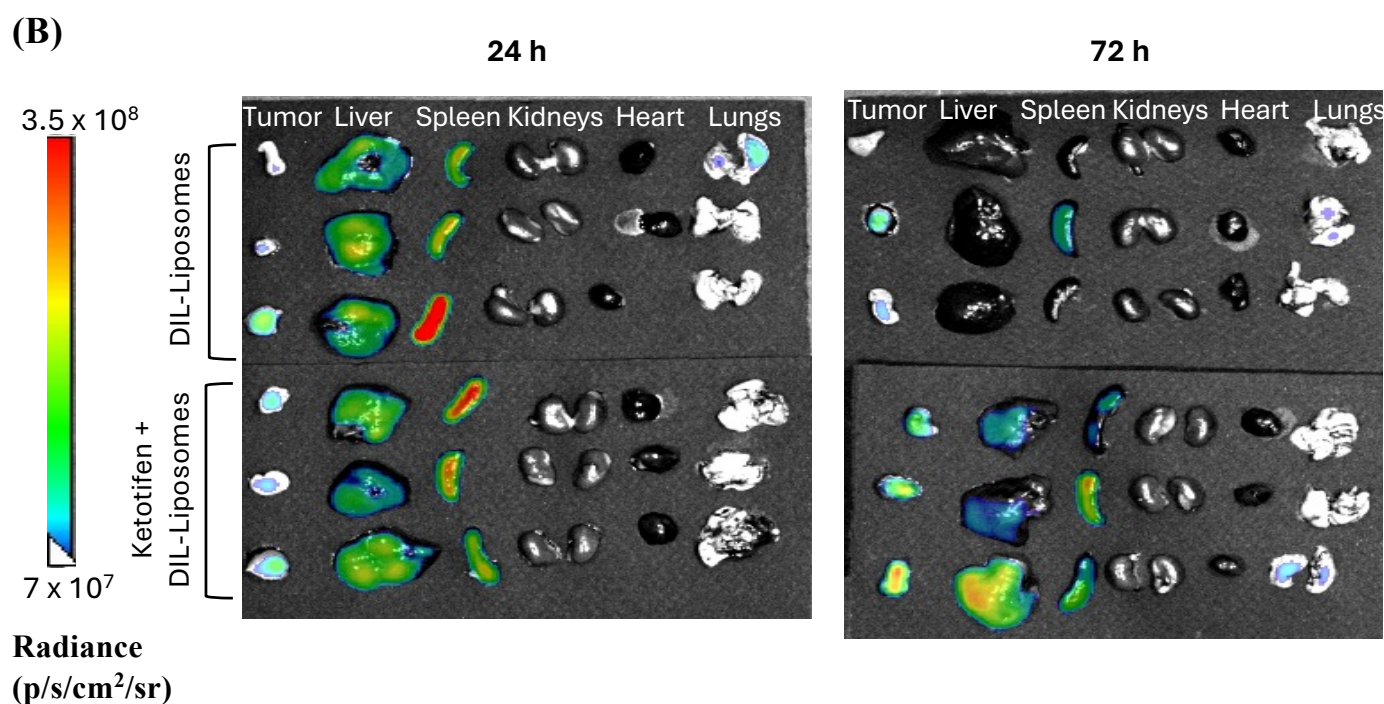

Supplement: Supplementary file 2 — Supplementary material 2 [file mmc2.pdf]

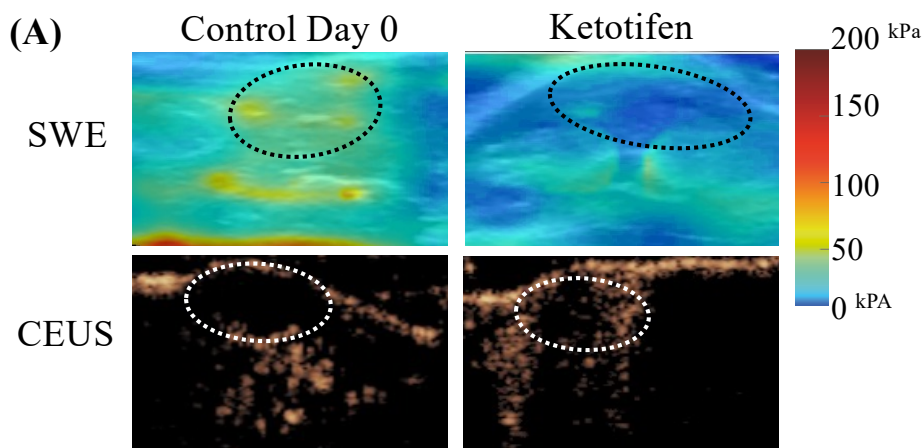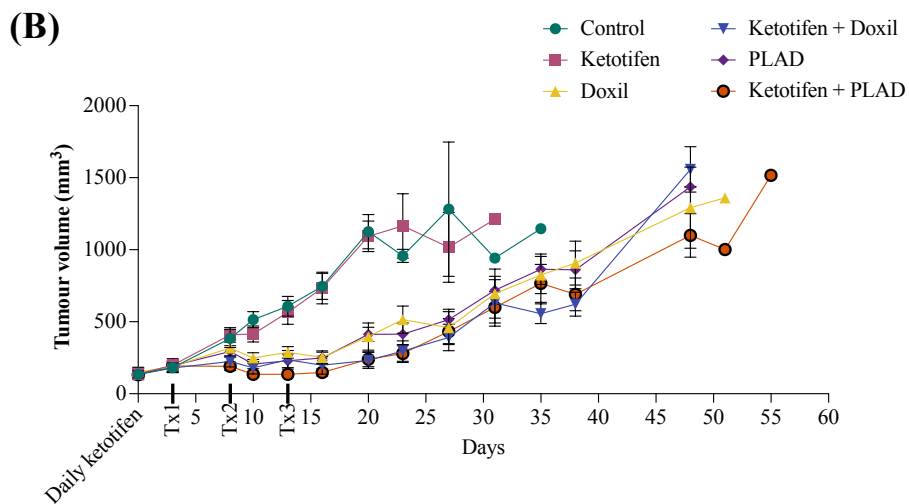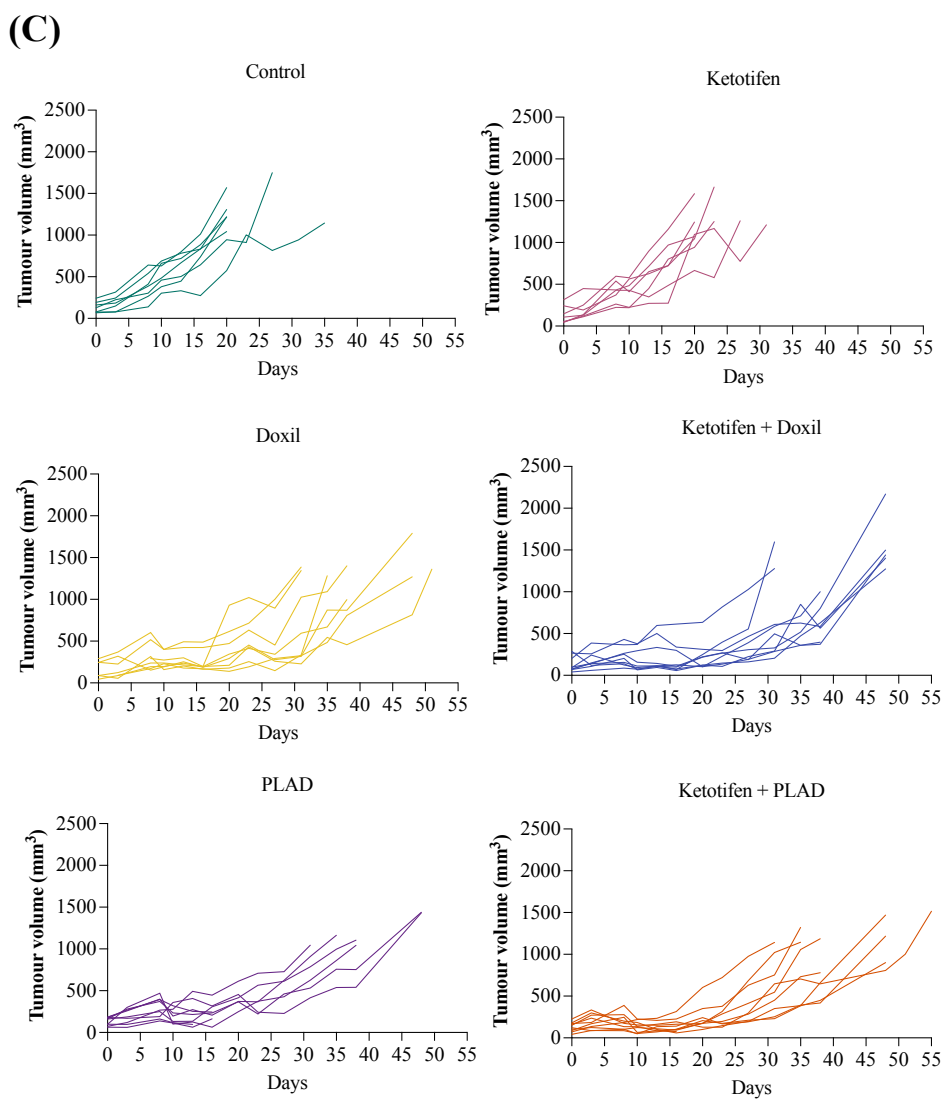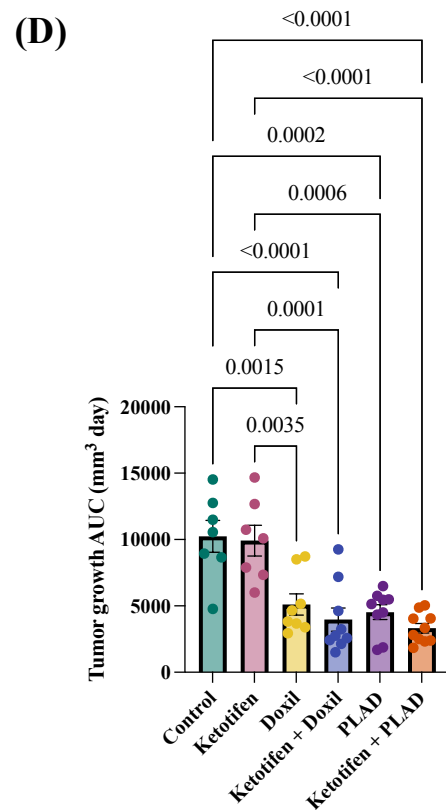

Supplement: Supplementary file 3 — Supplementary material 3 [file mmc3.pdf]

(A)

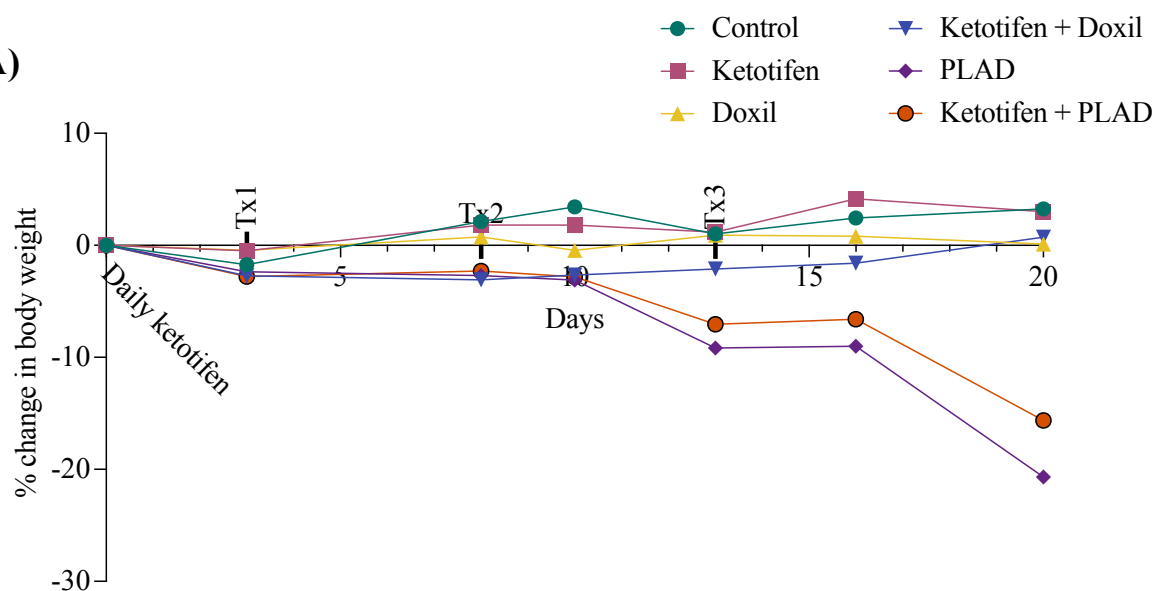

(B)

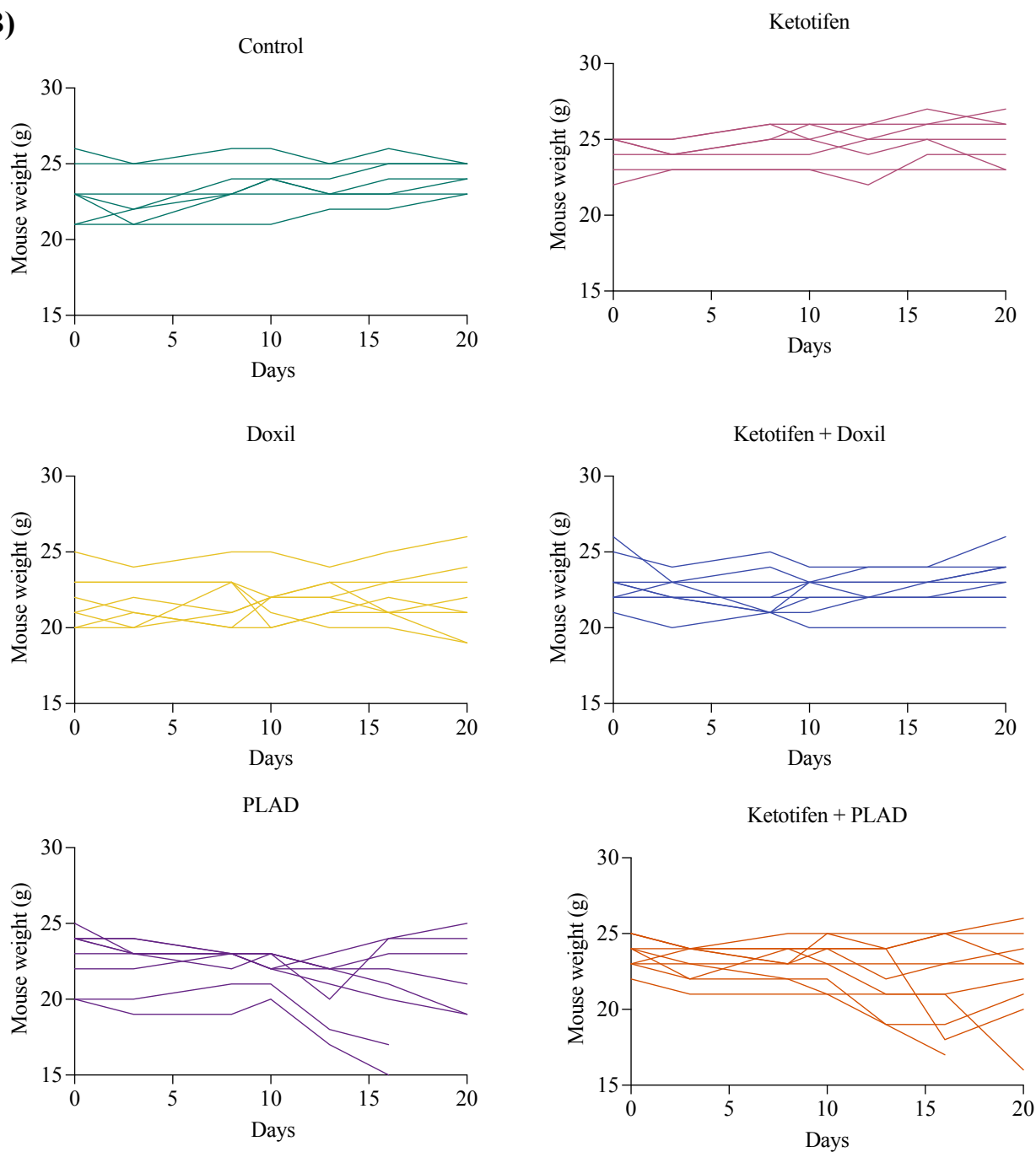

Supplement: Supplementary file 4 — Supplementary material 4 [file mmc4.pdf]

(A)

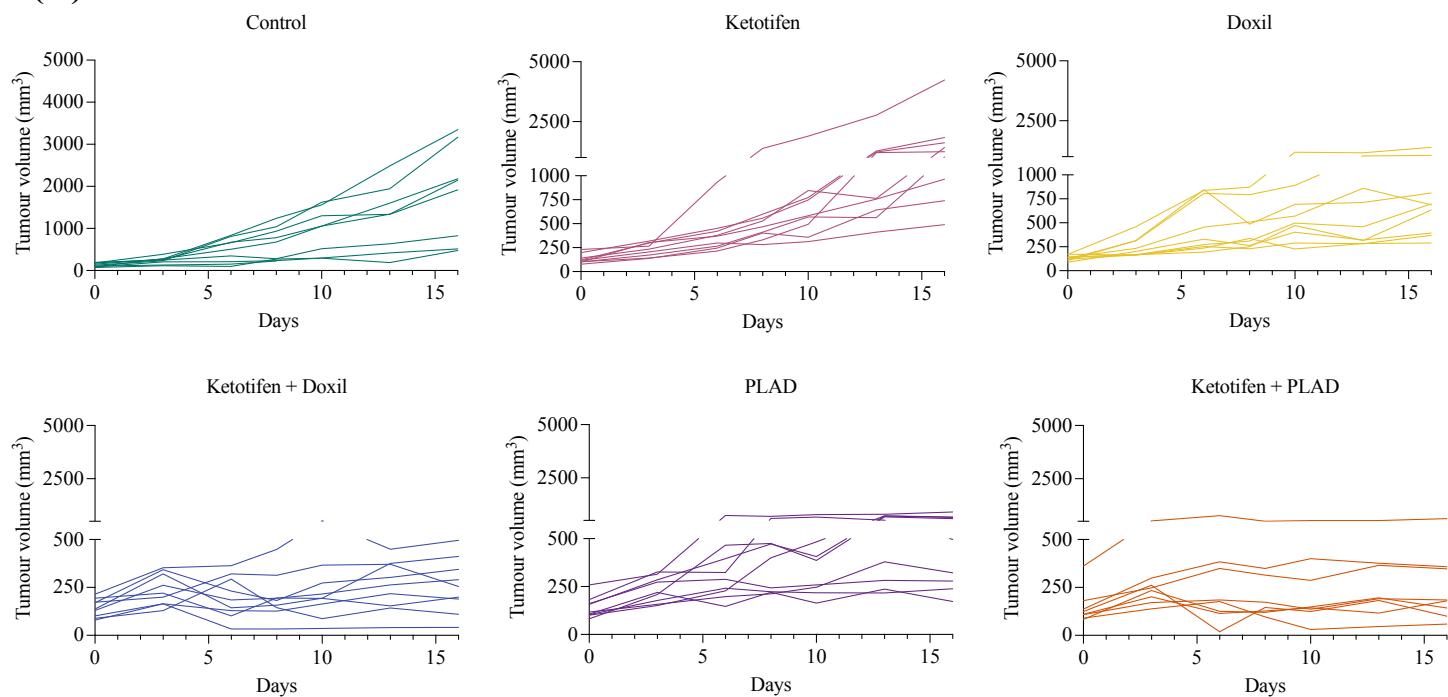

(B)

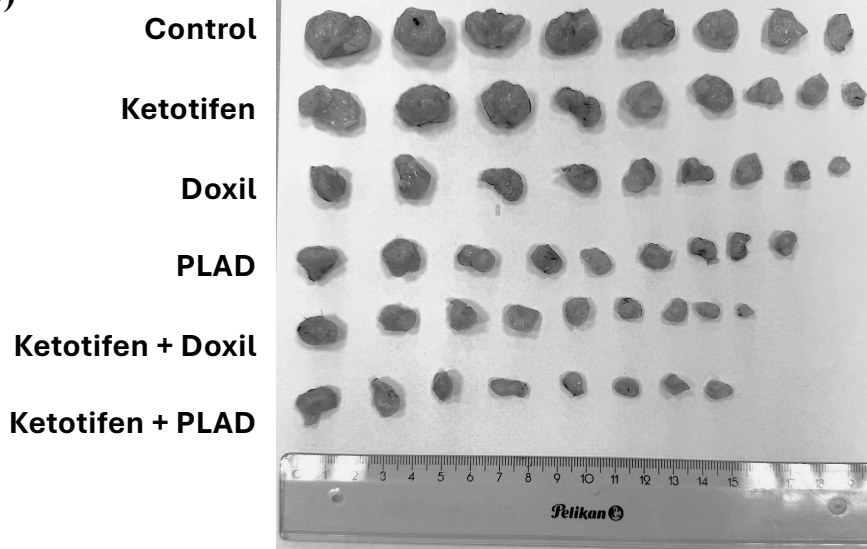

(C)

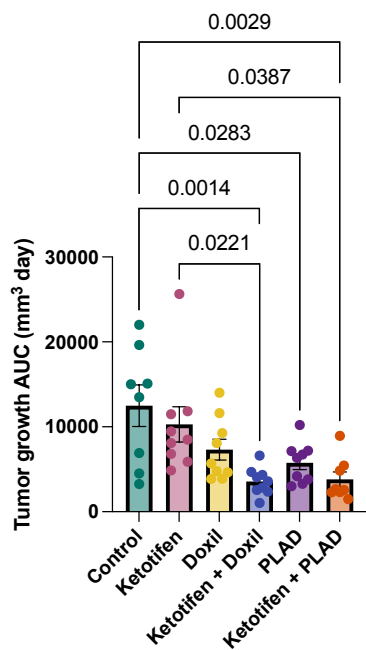

Supplement: Supplementary file 5 — Supplementary material 5 [file mmc5.pdf]

(A)

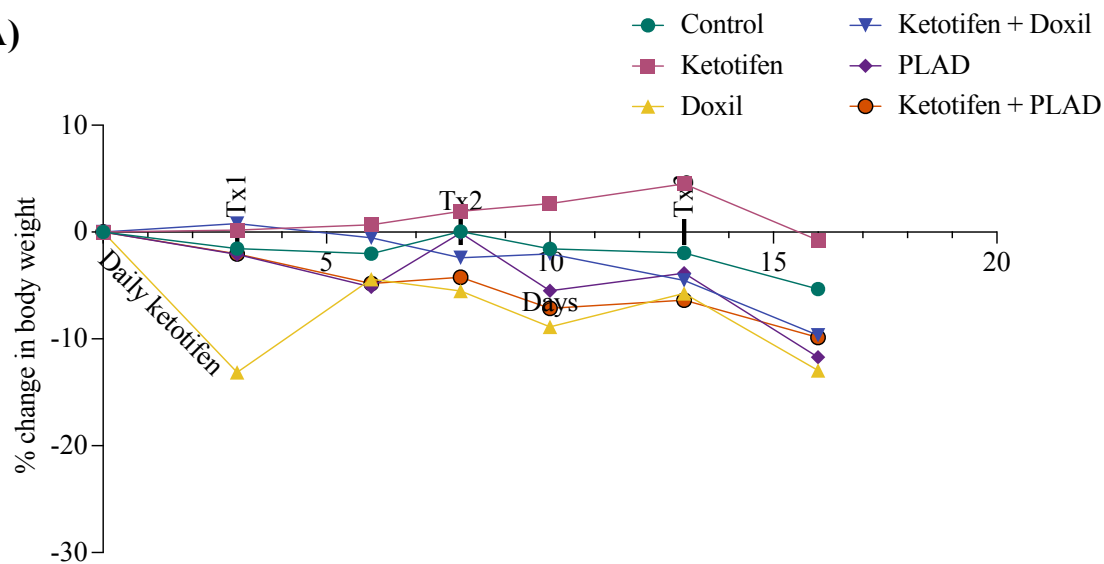

(B)

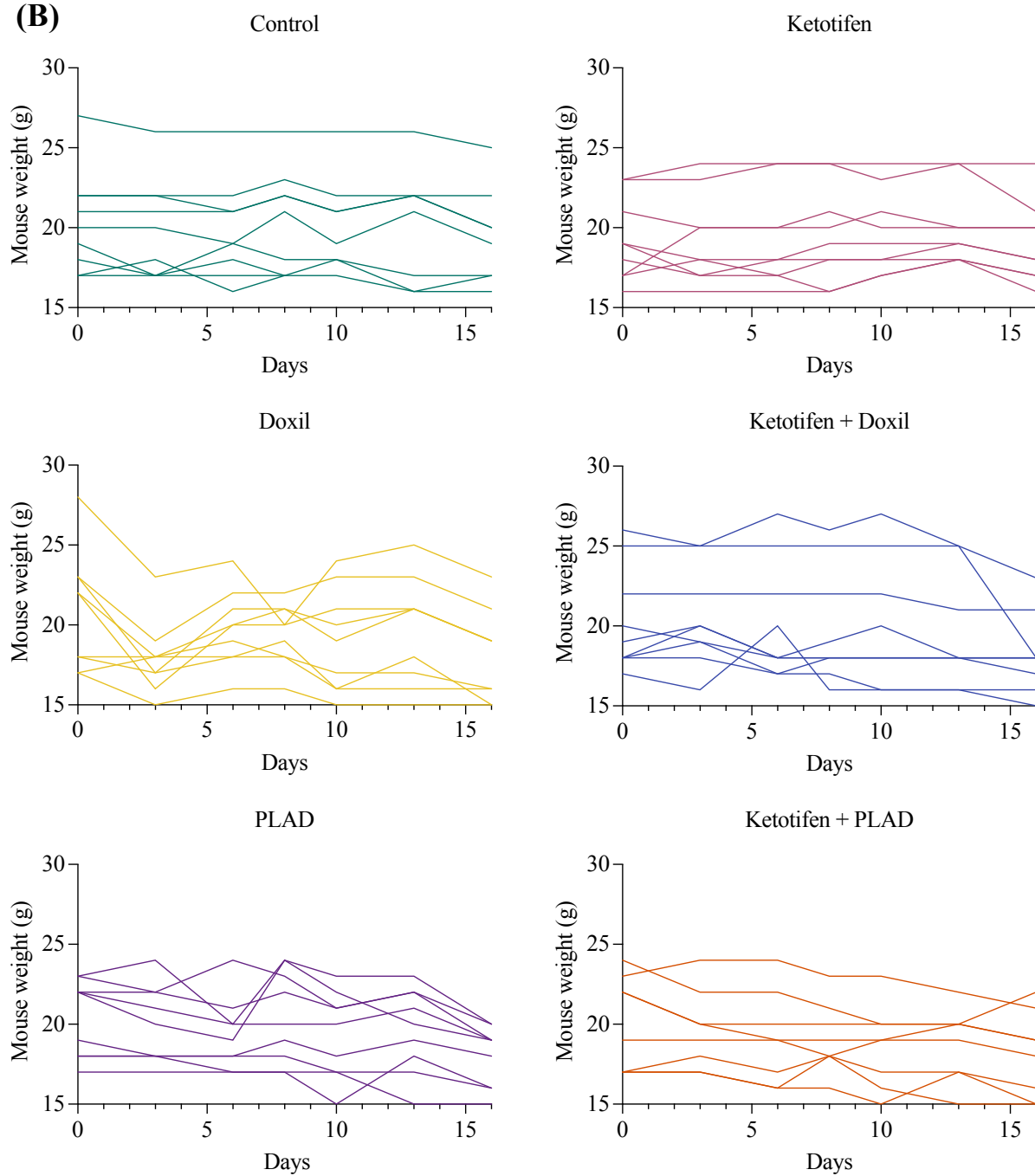

Supplement: Supplementary file 6 — Supplementary material 6 [file mmc6.pdf]

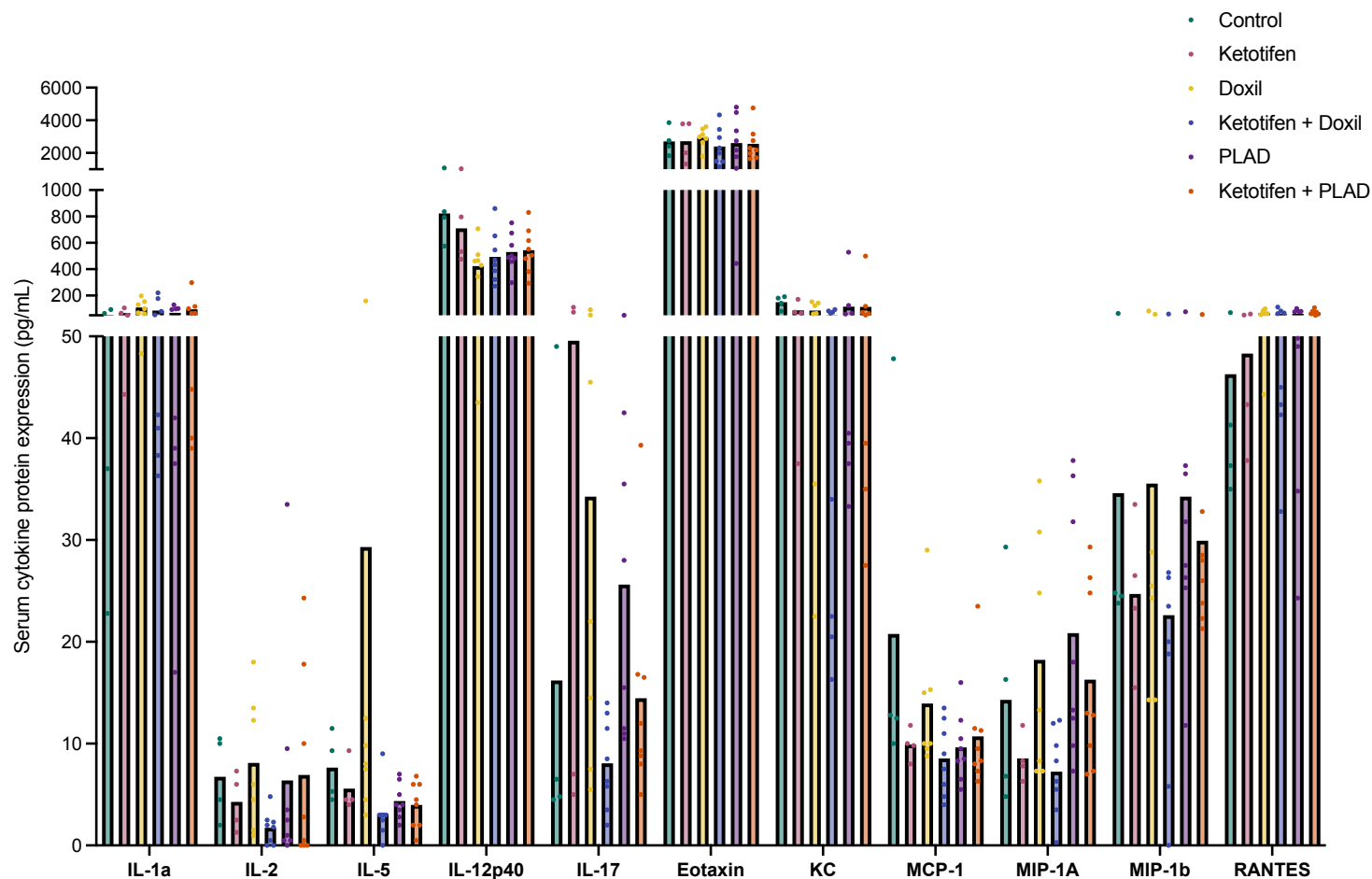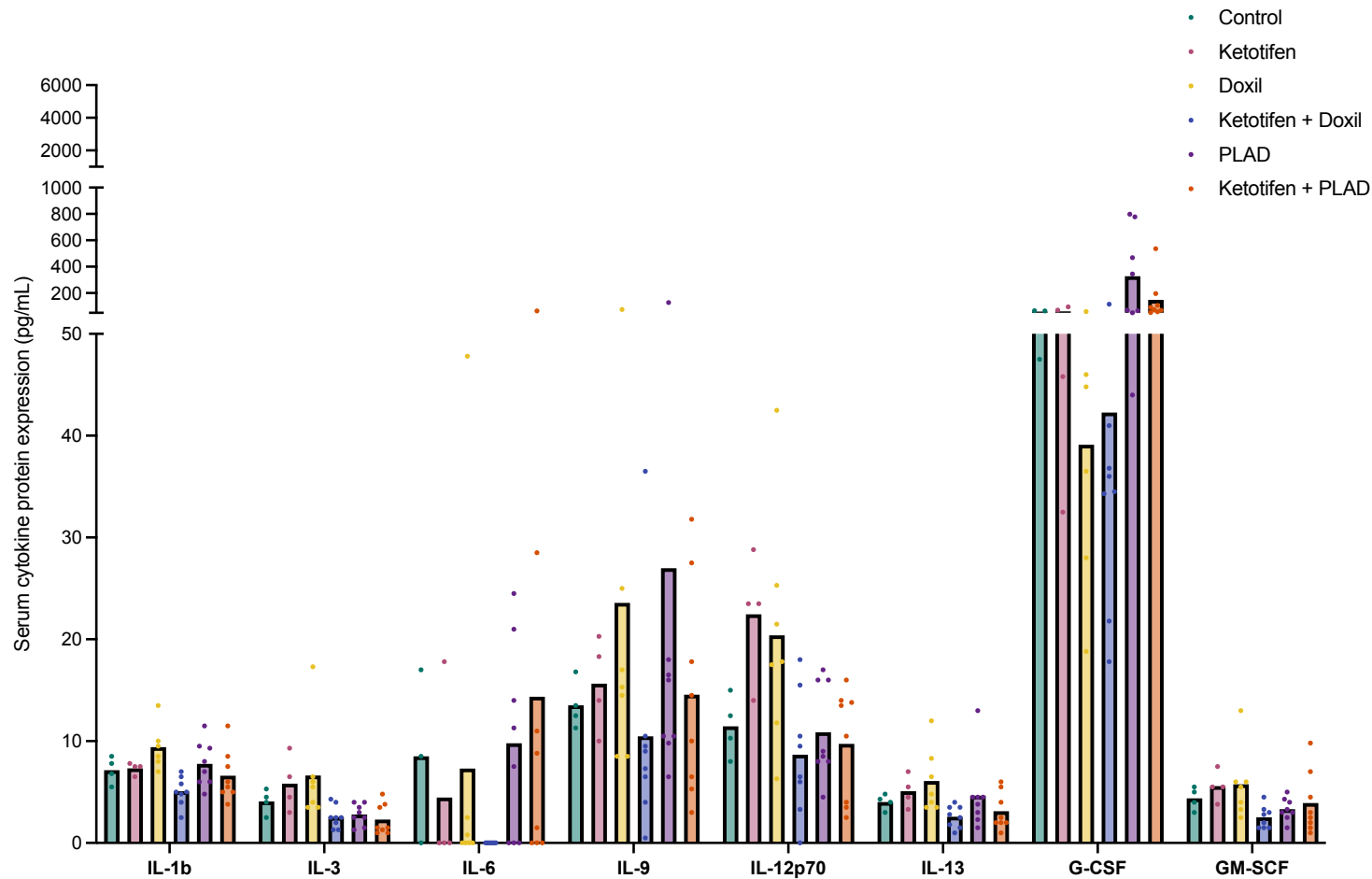

Supplement: Supplementary file 7 — Supplementary material 7 [file mmc7.pdf]

**(A)**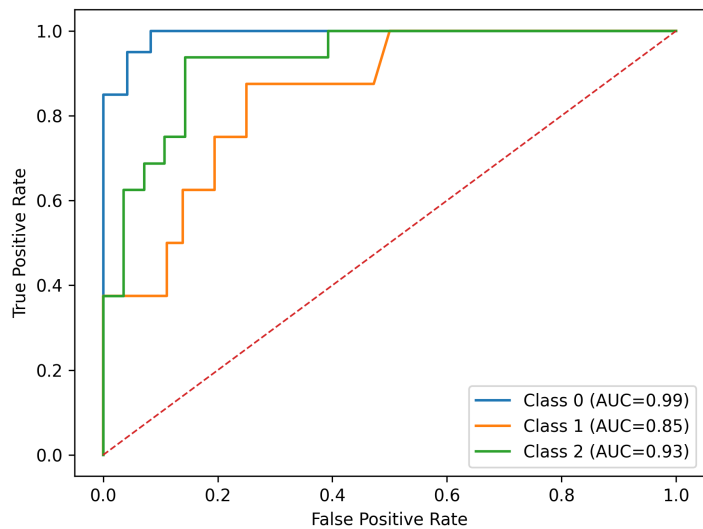**(B)**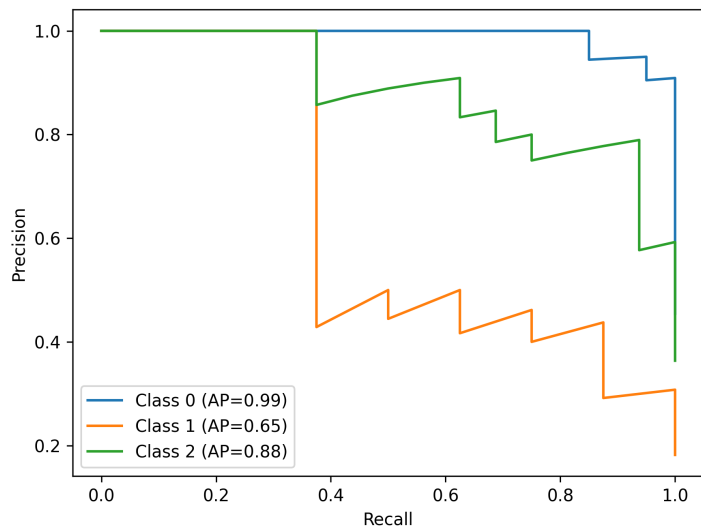**(C)**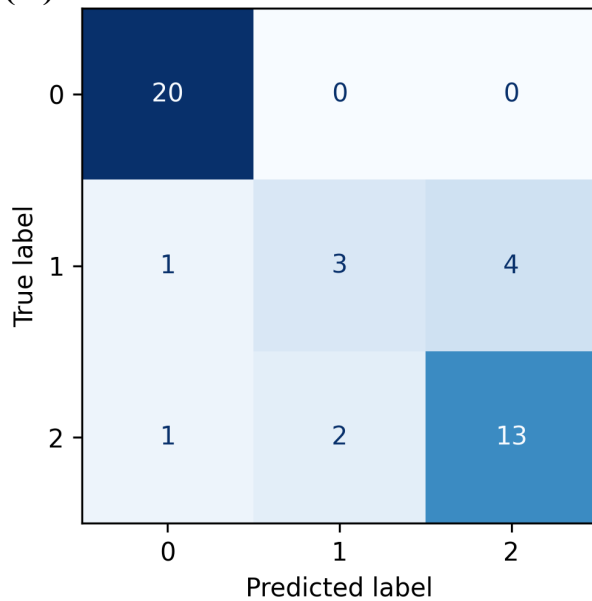

Supplement: Supplementary file 8 — Supplementary material 8 [file mmc8.pdf]
