# Supplementary material for: Enhancing nanomedicine efficacy in KPC pancreatic tumors through ketotifen-mediated tumor microenvironment remodeling
Source: J Control Release. 2026 Feb 10;390:114541. doi: 10.1016/j.jconrel.2025.114541 (PMC12888560; doi:10.1016/j.jconrel.2025.114541)
Supplement: Supplementary file 9 — Supplementary material 9 [file mmc9.pdf]

Supplementary Table S1: Primer sequences for qPCR		
Target gene	Forward	Reverse
IL-4	AACGAGGTCACAGGAGAAGG	TCTGCAGCTCCATGAGAACA
IFN-γ	ATGAACGCTACACACTGCATC	CCATCCTTTTGCCAGTTCCTC
TNF-α	CTGAACTTCGGGGTGATCGG	GGCTTGTCACTCGAATTTTGAGA
IL-10	GCTCTTACTGACTGGCATGAG	CGCAGCTCTAGGAGCATGTG
